# Wool Keratin Nanoparticle-Based Micropatterns for Cellular Guidance Applications

**DOI:** 10.1021/acsanm.2c03116

**Published:** 2022-10-04

**Authors:** Dagmara J. Trojanowska, Giulia Suarato, Clarissa Braccia, Andrea Armirotti, Fabrizio Fiorentini, Athanassia Athanassiou, Giovanni Perotto

**Affiliations:** † Istituto Italiano di Tecnologia, Smart Materials Group, Via Morego, 30, 16163Genova, Italy; ‡ Department of Materials Science, University of Milano-Bicocca, via R. Cozzi 55, 20125Milan, Italy; § Istituto Italiano di Tecnologia, Translational Pharmacology Facility, Via Morego, 30, 16163Genova, Italy; ∥ Istituto Italiano di Tecnologia, Analytical Chemistry Facility, Via Morego, 30, 16163Genova, Italy

**Keywords:** biointerfaces, laser desorption/ionization, molecule, leveraged, keratin

## Abstract

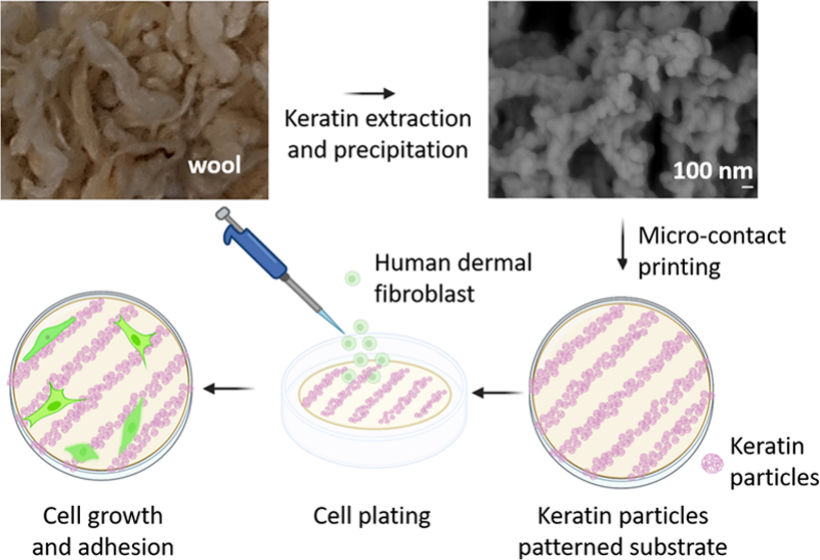

The waste stream of low-grade wool is an underutilized source of keratin-rich materials with appropriate methods for upcycling into high value-added products still being an open challenge. In the present work, keratins were precipitated from their water solution to produce hierarchical keratin particles via isoelectric precipitation. Matrix-assisted laser desorption/ionization coupled with time-of-flight tandem mass spectrometry analysis (MALDI-TOF/TOF MS/MS) showed the presence of the amino acid sequence leucine–aspartic acid–valine (LDV) in the extracted keratin. This well-known cell adhesion motif is recognized by the cell adhesion molecule α_4_β_1_ integrin. We showed that keratin particles had this tripeptide exposed on the surface and that it could be leveraged, via patterns obtained with microcontact printing, to support and facilitate dermal fibroblast cell adhesion and direct their growth orientation. The zeta potential, isoelectric point, morphological structures, chemical composition, and biocompatibility of keratin particles and the influence of the surfactant sodium dodecyl sulfate (SDS) were investigated. An appropriate ink for microcontact printing of the keratin particles was developed and micron-sized patterns were obtained. Cells adhered preferentially to the patterns, showing how this strategy could be used to functionalize biointerfaces.

## 1 Introduction

As the main component of wool, hair, nails, hooves, feathers, and horns, keratin is one of the most abundant structural proteins that occurs in nature. More than 2.5 million tons of wool is produced annually, of which 60% goes into apparel. However, a significant part of it ends up as a waste stream, including the low grades and trimmings obtained from slaughterhouses that do not enter the textile industry or fabric leftovers from the textile manufacturing process.^1,2^ Since these wastes have only few and limited applications (including low-quality animal feed, fertilizers, or biodegradable surfactants), the majority often ends up accumulated in landfills, buried, or disposed of by incineration. These actions are still practiced despite environmental hazards, including leachate and gaseous emissions, severe pressure of land scarcity (wool in landfills does not readily degrade), or toxic chemicals production.^3^ Wool is indeed comprised of 95% keratin proteins by weight and 5–10 mol % of the amino acids of keratins are sulfur-bearing cysteine residues.^1,4^ Therefore, finding new methods to provide value to the keratin contained in wool waste will allow to transform an underutilized biomass into high value-added products, improving the environmental and economical sustainability of the value chain. One of the most challenging obstacles to overcome is developing a simple and cheap procedure of keratin extraction due to its inherent high cross-linking. Keratins are structural proteins with high strength and stability thanks to the intra- and intermolecular interactions provided by the extensive hydrogen bonding and the disulfide bridges among the cysteine residues.^2^ Disulfide bonds permanently bind peptide chains, thus resulting in fundamental building of the keratin molecular architecture and, ultimately, in determining the material properties. The cortex of wool is the stress-bearing component, and it is composed of spindle-shaped cells of intermediate-filament proteins (IFPs) with a predominantly α-helical structure embedded in an amorphous high-sulfur protein matrix of β-keratin sheets of keratin-associated proteins (KAPs) that are rich in sulfur or glycine/tyrosine.^5^ Some IFPs, the hard α-keratins of wool, contain type I microfibrillar component 8C-1 and some contain type II microfibrillar component 7C, and these proteins are disulfide-bonded in the wool fiber to rich sulfur proteins in the KAP amorphous matrix.^5^ This structure makes keratin proteins resistant to physical, chemical (they are insoluble in water, weak acids, organic solvents, and resistant to chemical degradation), or environmental factors.^5^ To solubilize wool and subsequently extract keratins, it is necessary to disrupt the complex keratin structure. In the literature, several methods are reported for keratin extraction, with the most relevant being reduction,^6−8^ sulfitolysis,^6,9^ alkali treatment,^6^ oxidation,^10^ microwave irradiation,^11^ steam explosion,^12^ and dissolution in ionic liquids.^13^ We have run selected extractions to choose the most economical and environmentally friendly method and to provide a benchmark for keratin characterization (i.e., evaluation of yield, molecular weight, zeta potential, Fourier-transform infrared spectroscopy (FTIR), biocompatibility, and amino acid analysis) since the literature has some inhomogeneity.

There were many attempts to reutilize keratin in several fields of applications as a result of its versatile properties, including biocompatibility, biodegradability, natural abundance, and the presence of various functional groups. This natural material was converted into films, powders, hydrogels, foams, dressings, or scaffolds to be employed as a biosorbent for the removal of heavy metals^4^ or dye from aqueous solutions,^14^ as packaging material^15^ or as active and structural components in cosmetics and tissue engineering (cell culture growth, wound healing, nerve, and bone regeneration),^16^ surfactant,^17,18^ and recently into more technological applications like electronics.^18^

Micro- and nanoparticles of protein biomaterials, and of keratin in particular, can find interesting applications since they can be applied as building blocks or as drug delivery systems.^4^ The significant advantages of using naturally occurring materials in particle preparation are associated with their biodegradability, nontoxicity, relatively easy preparation, and high stability in biological fluids and during storage.^19^ Micro- and nanoparticles have indeed a high surface area per volume ratio, with numerous functional groups exposed on the surface that dictate their interaction with the environment. For example, keratin particles with different functional groups on their surface showed different interactions with mucosae glycoproteins.^20^ There are several methods for protein precipitation and preparation of protein particles, such as salting out, isoelectric precipitation, emulsification, desolvation, coacervation, mixing with nonionic hydrophilic polymers, and electrospraying technique.^20,21^ Keratin particles were obtained using the neutralization method,^22^ by decreasing the pH of keratin solution to its isoelectric point,^4,23−26^ via the emulsion diffusion method,^27^ electrospraying,^28^ enzymatic hydrolysis, and ultrasonication,^29^ desolvation and subsequent cross-linking with glutaraldehyde,^30^ via the precipitation method mediated by the phase separation of keratin and poly(vinyl alcohol)^20^ or ethylene glycol.^31^ Several studies have demonstrated that keratin particles were successful in removing Cu(II)^4^ from contaminated water and as drug delivery carriers,^20,26,31^ and presented antimicrobial,^23^ antioxidant, and anticancer activities.^24^ However, some of the reported methods required several experimental steps or sophisticated equipment; therefore, a fast and simple precipitation approach appears more promising.

In the past decade, keratin gained interest in the biomedical field not only thanks to its inherent biocompatibility but also due to its amino acid composition. In fact, keratin chains contain the amino acid sequence leucine–aspartic acid–valine (LDV), a well-known cell adhesion motif: this tripeptide is recognized by the cell adhesion molecule α_4_β_1_ integrin,^31−33^ which can be found at the membrane of a plethora of cells (leukocytes, macrophages, fibroblasts, smooth muscle cells, and endothelial cells).^32^ More specifically, the β_1_ integrin subunit plays a crucial role as a mechanosensory receptor in dermal fibroblasts, regulating tissue homeostasis and skin wound healing.^34^ This may imply that keratin can potentially act as a suitable tissue regeneration template. Cellular contact guidance is a phenomenon in which cells preferentially align to substrate micropatterns in a highly length-scale-dependent manner. The specific cell alignment in the direction of topographical or biochemical cues plays a crucial role in various physiological contexts, such as biological tissue formation.^35^ We aimed at studying if our keratin particles containing LDV domains would be able to facilitate cell adhesion, and if our keratin-based micropatterns could sustain the cell growth process and even induce a preferential alignment. By leveraging on both the polypeptide sequence and the nanoparticulate architecture, the utilization of keratin micropatterns could become an appropriate strategy for directing cell growth and, ultimately, tissue regeneration.

Microcontact printing (MCP) allows the fabrication of controllable substrates patterned with proteins for the study of specific cellular and tissue processes in which either spatial constraints or a precise directionality are necessary. It is important to note that the methods available in the literature mainly describe the MCP of protein solutions^35,36^ and the publications are minimal on the printing of protein-based and other particles. For instance, Balci and co-workers^37^ describe the printing process of *tobacco mosaic virus* particles, while the report of Xu et al. introduces the MCP method of silica nanoparticles.^38^

In the present work, keratin was extracted from wool using various protocols. Two protocols, i.e., sulfitolysis and reduction with cysteine, were selected for the keratin nanoparticle preparation based on the high yield and molecular weight of the products. The keratin nanoparticles were prepared by lowering the pH of aqueous keratin solutions to their isoelectric point with hydrochloric acid. At this pH, the net charge of the proteins approaches zero; the repulsive electrostatic forces are reduced, causing keratin aggregation and precipitation (isoelectric precipitation) into nanoparticles.^25^ Micropatterns composed of keratin particles printed on glass substrates were prepared using MCP, to assess if keratin particles could be able to offer binding sites to cell surface ligands, thus promoting cell adhesion,^31^ and if their patterning could be used to direct cell growth.

## 2 Experimental Section

### 2.1 Materials

Merino wool fibers were kindly provided by Olimpias s.r.l. (Ponzano Veneto, TV, Italy). Acetone (98.5%), methanol (99.8%), sodium metabisulfite, sodium bisulfite (≥58.5% (SO_2_)), 2-mercaptoethanol (99%), l-cysteine (98.5%), sodium sulfide (98%), urea (≥98%), Trizma hydrochloride (≥99.0%), hydrochloric acid (37%), sodium hydroxide (98%), sodium dodecyl sulfate (SDS, 98,5%), FITC-albumin, and methylene blue were purchased from Sigma-Aldrich. Polydimethylsiloxane (PDMS) (Sylgard 184 Silicone Elastomer Kit) was purchased from Dow Chemical Co. Milli-Q water (resistivity 18.3 MΩ) was used for all of the processes.

### 2.2 Methods

#### 2.2.1 Keratin Extraction

The wool fibers were first immersed in a 1:1 solution of methanol and acetone overnight and then thoroughly rinsed with Milli-Q water three times. The cleaned fibers were allowed to dry overnight in an oven at 30 °C. According to the adapted protocols from the literature, each gram of pretreated wool was immersed in 20 mL of different aqueous solutions containing the following reagents:

1. sodium hydroxide solution with a concentration of 1.5 wt %,^6^
2. 0.5 M sodium sulfide,^8^
3. 1.66 M 2-mercaptoethanol, 0.2 M Tris-HCl, 0.5 g SDS per 1 g of wool used, and 8 M urea,^6^
4. cysteine 10 wt % based on the wool weight, the pH of the solution was adjusted to 10.5 using 50% NaOH solution, 0.5 g SDS per 1 g of wool, and 8 M urea,^7^
5. 0.5 M sodium bisulfite, 0.5 g SDS per 1 g of wool, and 8 M urea,^6^
6. 0.2 M sodium metabisulfite, 0.5 g SDS per 1 g of wool, and 8 M urea.^9^

The extractions were performed under continuous stirring at 70 °C for 24 h (except extraction 1 that was carried out for 3 h). Then, the solution was filtered through a 40-mesh stainless steel grid. The filtrate was subsequently dialyzed against Milli-Q water using a dialysis tubing (molecular weight cut-off, MWCO, 6000–8000 Da) for 3 days with six water changes. The resulting solution was centrifuged (two times, at 9000 rpm and 4 °C for 20 min) to remove fiber residues. The protein concentration was measured by drying a known volume of the solution and the process yield was calculated according to eq 1. To investigate the effect of SDS on keratin extraction, the selected procedures (4 and 6) were also carried out without using SDS
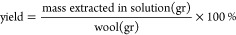
1

#### 2.2.2 Sodium Dodecyl Sulfate-Polyacrylamide Gel Electrophoresis (SDS-PAGE)

SDS-PAGE was performed to characterize the extracts from wool. SDS-PAGE, 4–15% Mini-PROTEAN TGX Precast Protein Gel (Bio-Rad Laboratories, Inc.), was used to resolve proteins at 200 V for 40 min. A wide-range molecular weight (10–250 kDa) marker (Precision Plus Protein Dual Color Standards, Bio-Rad Laboratories, Inc.) was run along with the proteins. Freeze-dried keratins dispersed in Milli-Q (10 mg/mL) were added to an appropriate volume of 4× laemmli sample buffer (Bio-Rad Laboratories, Inc.) with 200 mM dithiothreitol (DTT, Bio-Rad Laboratories, Inc.) and heated at 90 °C for 5 min to induce denaturation of the protein samples. The gel was colored with 0.1 wt % Coomassie Brilliant Blue R-250 (Bio-Rad Laboratories, Inc.) in a mixture of methanol/acetic acid/water (50:10:40, in volume) for 60 min and decolored in methanol/acetic acid/water (50:10:40, in volume) overnight.

#### 2.2.3 Amino Acid Analysis

Samples were derivatized with iodoacetic acid in ammonium bicarbonate and hydrolyzed in 6 M HCl at 110 °C under nitrogen. The hydrolysates were derivatized with orthophthalaldelyde (OPA) and 9-fluorenyl-methyl-chloroformate (FMOC) and analyzed by Jasco X-LC Amino Acid Analyzer with a fluorescence detector (excitation/emission at 340:446 nm for OPA amino acids and excitation/emission at 268:308 nm for FMOC amino acids).^39^

Quantitative amino acid composition was determined by external standard calibration using an Amino Acid Standard (Agilent) and CM-Cysteine (Sigma-Aldrich).

#### 2.2.4 In-Gel Digestion and Matrix-Assisted Laser Desorption/Ionization Coupled with Time-of-Flight Tandem Mass Spectrometer Analysis (MALDI-TOF/TOF MS/MS)

**Figure 1:**
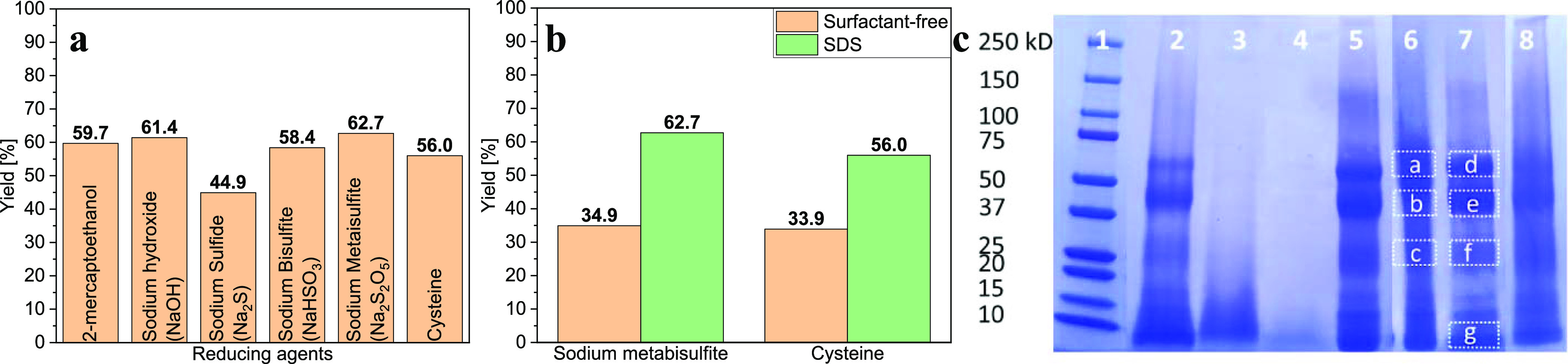
(a) Effects of different extraction procedures on keratin extraction yield. (b) Effects of surfactant on keratin extraction yield. (c) SDS-PAGE patterns of protein standard (1), keratin extracted from wool using 2-mercaptoethanol (2), sodium hydroxide (3), sodium sulfide (4), sodium bisulfite (5), sodium metabisulfite (6), sodium metabisulfite + SDS (7), and cysteine (8). Highlighted bands (dashed boxes) were used for in-gel digestion.

We performed an in-gel digestion of the bands highlighted in Figure 1C, lines 6 and 7 (a–h bands highlighted by dashed boxes), following a well-established protocol already described.^40^ Briefly, each band was destained twice for 10 min using 100 μL of 50% acetonitrile (MeCN) and 50% digestion buffer (50 mM NH_4_HCO_3_, pH 8). Disulfide bonds were reduced by incubating the samples at 56 °C for 1 h with 100 μL of 10 mM dithiothreitol, dissolved in 50 mM ammonium bicarbonate. After the removal of the reducing solution, gel pieces were washed with digestion buffer. Cysteine residues were then alkylated by adding 100 μL of 55 mM iodoacetamide dissolved in digestion buffer. After incubation in the dark for 45 min, the alkylating solution was discarded, and gel pieces were washed as in the previous step. Gel pieces were then dehydrated with 100 μL of MeCN. Protein digestion was performed by adding 1 μg of Trypsin (from porcine pancreas, proteomics grade, code number: T6567 from Sigma-Aldrich) and incubating the samples overnight at 37 °C. The solutions containing the peptides were collected in new Eppendorf tubes. Gel pieces were then incubated for 10 min with 50 μL of extraction solution (30% MeCN + 3% trifluoroacetic acid, TFA). After the incubation, the solution in each tube was collected and pooled with the corresponding initial peptide mixture. The last peptides extraction was performed by incubating the samples with MeCN for 10 min. As done in the previous step, the solution of the peptides was collected again and pooled with the previous one. The peptides were dried under vacuum. For MALDI-TOF/TOF MS/MS analysis, the dried peptides were dissolved in 30 μl of 3% MeCN + 0.1% TFA and then diluted 1:1 with matrix solution (α-cyano-4-hydroxycinnamic acid, HCCA, 5 mg/mL dissolved in 50% MeCN + 0.1% TFA). The mixture solution (1 μL) was spotted on a MALDI 96-target polished plate (Bruker Daltonics). Mass spectra were acquired in positive reflector mode using an ultrafleXtreme MALDI-TOF/TOF mass spectrometer (Bruker Daltonics) with automated fragmentation of selected ions. The mass range was set at 700–3500 Da for the MS mode and 40–1800 Da for the tandem mass (MS/MS) acquisition. Spectra were calibrated using a bovine serum albumin digest standard. Spectra for peptide and protein identification were searched against SwissProt database, taxonomy other mammalia, using the Mascot search engine. The parameters were set as follows: 50 ppm and 0.3 Da error tolerance for MS and MS/MS spectra, respectively; one missed cleavage; carbamidomethylation on cysteine residues as fixed modification; and oxidation on methionines as variable modification.

#### 2.2.5 Zeta Potential

The size and zeta potential of keratin solutions and keratin particles were characterized by DLS using a Malvern Zeta Sizer NanoZS. Three acquisitions per sample were recorded. The zeta potential was measured between –200 and +200 mV. The zeta potential measurements were conducted at pH values from 7 to 2 to determine the isoelectric potential.

#### 2.2.6 Keratin Particle Fabrication

The pH of keratin solutions (1 mg/mL) was adjusted from 5 to 2 using 2 N HCl. Precipitation of keratin into micro- and nanoparticles occurred when the pH was closest to the isoelectric point. Particles were collected after 24 h, purified by dispersion in Milli-Q water, and centrifuged for 10 min at 1000 rpm (two times). After purification, the particles were lyophilized. The particles were prepared starting from the following keratin solutions: keratin extracted via the sulfitolysis process (labeled KSPs) with and without SDS, and keratin obtained by reduction with cysteine (labeled KCPs) with and without SDS. The method was adapted from ref (24).^24^

Moreover, model drugs were encapsulated in keratin particles. Methylene blue and FITC-albumin were selected because both give a specific color to the loaded particles and were successfully encapsulated in our previous work.^20^ To evaluate the encapsulation efficiency, keratin particles were prepared from keratin solutions (as above) where the model drug was dissolved. The precipitate was washed with Milli-Q water. The encapsulation efficiency was characterized by comparing the absorption peak of the solution with the concentration of drugs used for encapsulation with an absorption peak of supernatant after isoelectric precipitation. The absorbance peak was measured using a UV–visible spectrophotometer (Cary 6000i-Varian). The encapsulation efficiency of FITC-albumin was determined spectrophotometrically by correlating the absorbance at 495 nm, while that of methylene blue at 669 nm.

#### 2.2.7 FTIR Characterization

Infrared spectra were measured in the attenuated total reflectance (ATR) configuration coupling a MIRacle ATR (PIKE Technologies) to a Fourier transform infrared (FTIR) spectrometer (Vertex 70V, Bruker, Germany). All spectra were recorded in the range from 4000 to 600 cm^–1^ with a resolution of 4 cm^–1^, accumulating 32 scans.

#### 2.2.8 Electron and Optical Microscopy

The morphology of keratin particles was analyzed using a JEOL JSM-7500FA SEM, an analytical field-emission scanning electron microscope (SEM), with an accelerating voltage of 5 kV. The particles were placed on a conductive carbon adhesive tape and coated with a 10 nm gold layer to allow imaging. The diameters of the particles and aggregates were obtained by the average diameter of 30 particles or agglomerates measured using ImageJ (https://imagej.nih.gov).

The 2D and 3D acquisition of the surface morphology of the patterns was obtained by an optical profilometer zeta-20 by ZETA. The 3D image was obtained with ProfilmOnline.

#### 2.2.9 Generation of the Silicon Master Molds

##### 2.2.9.1 Silicon Stripes 50–50 μm Gap Patterning Procedure

The 50–50 μm pattern was written by a Heidelberg DWL66FS laser system on an AZ5214 resist, which was previously spinned onto the wafer at 4000 rpm and then baked at 120 °C for 1 min. The exposed resist was developed by the AZ726 developer for 1 min, the wafer was then cleaned via an oxygen plasma cleaner (for 60 s at 100 W). The remaining resist was completely removed in acetone, and the wafer was cleaned as mentioned before. Afterward, holes were etched in a Sentech SI500 ICP-RIE system by a three-step Bosch-like process at a 100 W ICP power and rate of 150 nm/cycle, leading to about 5.0 μm thickness in 30 cycles (Figure 1Sa,b). Lastly, the resist was completely removed in acetone and the wafer was cleaned with piranha solution (for 2 min and 30 s) and by an oxygen plasma cleaner (for 60 s at 100 W).

##### 2.2.9.2 Silicon Stripes 5–10 μm Gap Patterning Procedure

The 5–10 μm pattern was written by a Süss MicroTec MA6/BA6 Mask Aligner system on a MICROPOSIT S1813 resist, which was previously spinned onto the wafer at 4000 rpm and baked at 95 °C for 1 min. The exposed parts of the resist were developed by a MICROPOSIT MF-319 developer for 45 s, and the wafer was cleaned via an oxygen plasma cleaner (for 60 s at 100 W). The remaining resist was completely removed in acetone, and the wafer was cleaned as mentioned above. Afterward, holes were etched in a Sentech SI500 ICP-RIE by a three-step Bosch-like process at a 100 W ICP power and a rate of 150 nm/cycle, leading to about 4.9 μm thickness in 45 cycles (Figure 1Sc,d). Lastly, the resist was completely removed in acetone and the wafer was cleaned with an oxygen plasma cleaner (180 s at 100 W). The thicknesses of the patterned Silicon masters were observed under a Helios Nanolab 650 SEM and measured with a Veeco Dektak 150 profilometer to assess the presence of the stripes (Figure 1Sa–d).

#### 2.2.10 Preparation of PDMS Micropatterned Stamps (Replica Molding)

PDMS micropatterned stamps were prepared according to ref (36). Briefly, PDMS was obtained using a Sylgard 184 Silicone Elastomer Kit (Sylgrad 184 prepolymer and curing agent mass ratio 10:1). The master molds (see Section 2.2.7) were placed in the middle of a 150 mm diameter petri dish and covered with PDMS (∼40 g). The unpolymerized PDMS was degassed until all bubbles disappeared. Subsequently, the master molds covered with PDMS were cured at 65 °C for 1 h. After the curing time, one edge of PDMS attached to the master mold was released using a scalpel blade and the rest of the PDMS was gently peeled off. In the following step, the PDMS with the patterned surface facing up was cut into rectangular pieces 5 mm in width and 10 mm in length. The orientation of the stripes was parallel to the short edge of the PDMS rectangle. The PDMS micropatterned stamp surfaces were oxidized using O_2_ plasma (20 sccm, 120 s) to confer their hydrophilicity. Plasma oxidized PDMS stamps were submerged in Milli-Q water to prevent the PDMS surface from becoming hydrophobic again.

#### 2.2.11 Protein Microcontact Printing (MCP)

To fabricate the patterns, freeze-dried particles were dispersed in Milli-Q water, ethanol, or a mixture of ethanol and Milli-Q water (0.5, 1, and 5 mg/mL), sonicated (40 kHz frequency, FALC Instruments), and used as an ink for the MCP. The keratin-based ink (30 μL) was deposited on plasma oxidized PDMS stamps presenting two different patterns (see Section 2.2.7 and Section 2.2.8): 10 μm wide stripes spaced 5 μm apart (5–10) and 50 μm wide stripes spaced 50 μm apart (50–50) and two different types of keratin particles, KSPs and KCPs. In the inking step, the particles are randomly placed on the oxidized PDMS stamp. After the incubation time, the inked stamps were dried with N_2_ flow. In the next step, the stamps were wetted with 15 μL of ethanol and again dried with N_2_ flow (leaving a layer of ethanol on the surface of the stamp), placed on the glass coverslips with the patterned surface down (stamping step) and let adhere for different times (1–60 min). During the stamping step, the keratin-based ink solutions were transferred in organized stripes, mirroring the PDMS stamp pattern. The biocompatibility of the stripes, as well as the adhesion and morphology of the cells plated on various keratin patterns under study were subsequently evaluated.

#### 2.2.12 Assessment of the Biocompatibility of Keratin Particles and Control Experiments

Primary adult human dermal fibroblast (HDFa, Thermo Fisher Scientific) cells were cultured in T75 culture flasks in the presence of Fibroblast Growth Medium 2 (Sigma-Aldrich) supplemented with a pack containing fetal calf serum (0.02 mL/mL), basic fibroblast growth factor (recombinant human, 1 ng/mL), insulin (5 μg/mL), and pen/strep (1%) in an incubator set at 37 °C and with 5% CO_2_. Keratin particle extracts were prepared by dispersing lyophilized particles (KCPs + SDS, KCPs, KSPs + SDS, KSPs) in culture media at a concentration of 1 mg/mL. The media were then incubated for 24 h at 37 °C and 5% CO_2_ and subsequently used to treat the cells. To determine fibroblast viability, an MTS assay (tetrazolium salt, CellTiter 96AQ_ueous_ One Solution Cell Proliferation Assay, Promega) was conducted following the protocol previously established in our group.^34^ Briefly, fibroblasts were seeded in 24-well plates at a cell density of 5000 cells/cm^2^ and let attach overnight. Afterward, the medium was replaced with extraction one (500 μL per well) and the test was carried out for 24 and 48 h. At the desired time points, the medium was again changed with a fresh one and 25 μL of MTS reagent was added to each well. After 3.5 h of incubation at 37 °C and 5% CO_2_, optical densities at 490 nm were read by a plate reader. Results are reported as mean value ± standard error. A student’s *t*-test assuming unequal variances was conducted considering *p* < 0.01 (**).

In a second control experiment, aiming to assess the effect of the KSPs and KCPs on the general cell morphology, only the KPs without SDS were considered. Briefly, glass coverslips were coated with KP suspensions at a 1 mg/mL concentration and let dry to form a nonpatterned substrate, with randomly deposited particles and aggregates. The samples were sterilized under UV light for 30 min. HDFa cells were plated at a density of 2000 cells/cm^2^ and let grow for 72 h. The fixing and staining procedures were carried out as described in Section 2.2.14and the samples were analyzed via confocal microscopy.

#### 2.2.13 Assessment of the Stripe Biocompatibility

A live/dead staining assay was performed on four different striped patterns under study (5–10 KCPs; 50–50 KCPs; 5–10 KSPs; 50–50 KSPs). The patterned 13 mm glass coverslips were sterilized under UV light for 30 min and placed at the well bottoms of a 24-well plate. HDFa cells were seeded at a density of 7000 cells/cm^2^. This cell density was used because a rather dense cell population for statistical analysis is needed to assess the potential toxicity arising from direct contact between KPs and the fibroblasts. A higher number of cells could have led to the formation of a stratified cellular structure (especially at longer time points), which might have interfered with cell counting via image processing. Cells were let attach and grow on the patterned stripes for either 24 or 48 h in a humidified incubator at 37 °C and with 5% CO_2_, in the presence of Fibroblast Growth Medium 2 (Sigma-Aldrich) supplemented with a supplement pack containing fetal calf serum (0.02 mL/mL), basic fibroblast growth factor (recombinant human, 1 ng/mL), and insulin (5 μg/mL). Cells plated directly onto the tissue culture wells were considered as a control sample.

After 24 or 48 h, 1.5 μL of calcein-AM (4 mM solution in DMSO, Sigma-Aldrich) and 1 μL of ethidium homodimer (2 mM solution in DMSO, Sigma-Aldrich) were added to each well (containing 500 μL of supplemented media) and incubated for an additional 45 min in a humidified chamber at 37 °C and with 5% CO_2_.

Images were taken immediately after the incubation by a confocal microscope Nikon A1, equipped with a 20× objective and with 488 and 401 nm lasers. An average of 20 images per sample were acquired and used for the cell counting, performed with ImageJ (https://imagej.nih.gov) via the Cell Counter plugin.

#### 2.2.14 Assessment of Cell Morphology

The adhesion and morphology assessment of the cells was performed on four different striped patterns under study (5–10 KCPs; 50–50 KCPs; 5–10 KSPs; 50–50 KSPs). The patterned 13 mm glass coverslips were sterilized under UV light for 30 min and placed at the well bottoms of a 24-well plate. HDFa cells were seeded at a density of 2000 cells/cm^2^. In general, when focusing on the inner structure of a cell (i.e., actin filaments, focal adhesion points arrangements) dealing with a less “crowded” sample enables a more detailed identification of such structures. Based on our experience, 2000 cells/cm^2^ represent a fair condition for the cells to interact almost individually with the substrate, thus allowing us to specifically study the effect of the pattern. Cells were let attach and grow on the patterned stripes for 72 h in a humidified incubator at 37 °C and with 5% CO_2_ in the presence of Fibroblasts Growth Medium 2 (Sigma-Aldrich) supplemented with a supplement pack containing fetal calf serum (0.02 mL/mL), basic fibroblast growth factor (recombinant human, 1 ng/mL), and insulin (5 μg/mL). Cells plated directly onto 13 mm glass coverslips, nonpatterned, were considered as control samples.

After 72 h, cells were stained for actin fibers, nuclei, and focal adhesion points as follows. The samples were fixed in 4% paraformaldehyde in 1 × phosphate buffers saline (PBS) for 20 min and then washed twice with prewarmed 1 × PBS. Afterward, cells were permeabilized with 0.1% Triton X-100 for 8 min and washed twice with prewarmed 1 × PBS. A blocking solution of 10% normal goat serum (Abcam) was applied for 1 h. As primary antibody, vinculin monoclonal antibody produced in mouse (Sigma-Aldrich) was diluted 1:400 in the blocking solution and applied for 1 h. The cells were washed three times (5 min each) in prewarmed 1 × PBS. Double labeling was conducted by preparing a solution in 1 × PBS of secondary antibody (AlexaFluor488 Goat Anti-Mouse (IgG) secondary Antibody, Abcam) at 1:1000 dilutions, and AlexaFluor488 Phalloidin (Thermo Fisher) at 1:100 dilutions. The samples were stained for 1 h at room temperature, covered with an Al foil and then washed three times (5 min each) in prewarmed 1 × PBS. Lastly, the cell nuclei were stained with DAPI (4′,6-diamidino-2-phenylindole, dihydrochloride, 2.5 μg/mL, Thermo Fisher) for 5 min at room temperature in the dark, and then washed twice with prewarmed 1 × PBS.

The stained coverslips were mounted onto microscope glass slides with the mounting media Fluoromount-G (Thermo Fisher). Images were taken by a confocal microscope Nikon A1, equipped with a 60× objective and with 488 and 401 nm lasers.

## 3 Results and Discussion

### 3.1 Keratin Extraction

Keratins were extracted from wool using thermochemical treatments: sulfitolysis (sodium bisulfite and sodium metabisulfite), hydrolysis (sodium hydroxide), and reduction (2-mercaptoethanol, cysteine, and sodium sulfide) and were examined in terms of yield, molecular weight, and chemical structure.

The keratin yields for the different processes were determined gravimetrically by drying a known volume of keratin solution and the results are shown in Figure 1a. The most effective 24 h treatment of wool was the sulfitolysis process with an extraction yield of ∼63%, while the chemical treatments in alkaline conditions (NaOH or Na_2_S treatment where Na_2_S reacts in water to form hydrosulfate and hydroxyl ions, giving the reaction mixture a high pH^41^) had lower extraction yields, most probably because the peptide linkages were partially broken and were removed during purification.^25^

The presented results in Figure 1a (apart from sodium hydroxide and sodium sulfate treatments) were obtained with the use of the anionic surfactant (sodium dodecyl sulfate, SDS) since it was found that it substantially increases the yield of the obtained keratin. In general, surfactant application in the keratin extraction process enhances the stability of keratin solutions, prevents aggregation, accelerates the extraction, and positively influences the extraction yield.^42,43^ The surfactant utilization in our case nearly doubled the extraction yield, as shown in Figure 1b. This is probably because the surfactant forms a complex with the keratin, stabilizing keratin in the water solution and resulting in a higher yield. The formation of these complexes was observed to cause the removal by dialysis of the surfactant at a much slower rate than other low molecular mass compounds.^43^

SDS-PAGE was used to establish the molecular weight (MW) of keratin extracts; data are shown in Figure 1c. SDS-PAGE patterns show that the MW values of the keratins were influenced by the extraction protocol. Sodium hydroxide and sodium sulfide treatments produced prevalently low MW keratins, confirming the degradation of the proteins caused by peptide hydrolysis at high pH values.^26^ Consequently, the peptides with a molecular weight lower than 6000–8000 Da (the dialysis membrane molecular weight cut-off used for the purification) were removed during the dialysis process. The other methods resulted in products with a MW range of 6–65 kDa for wool keratin. These results are in agreement with refs (7, 10). The bands between 6 and 30 kDa indicate the presence of amorphous, low MW, high-sulfur keratins (11–26 kDa) and the glycine- and tyrosine-rich proteins (6–9 kDa) that are derived from the interfilament structures. On the other hand, the bands between 40 and 65 kDa correspond to the low-sulfur keratins originating from intermediate-filament proteins with a predominantly α-helical structure.^10,44^

**Figure 2:**
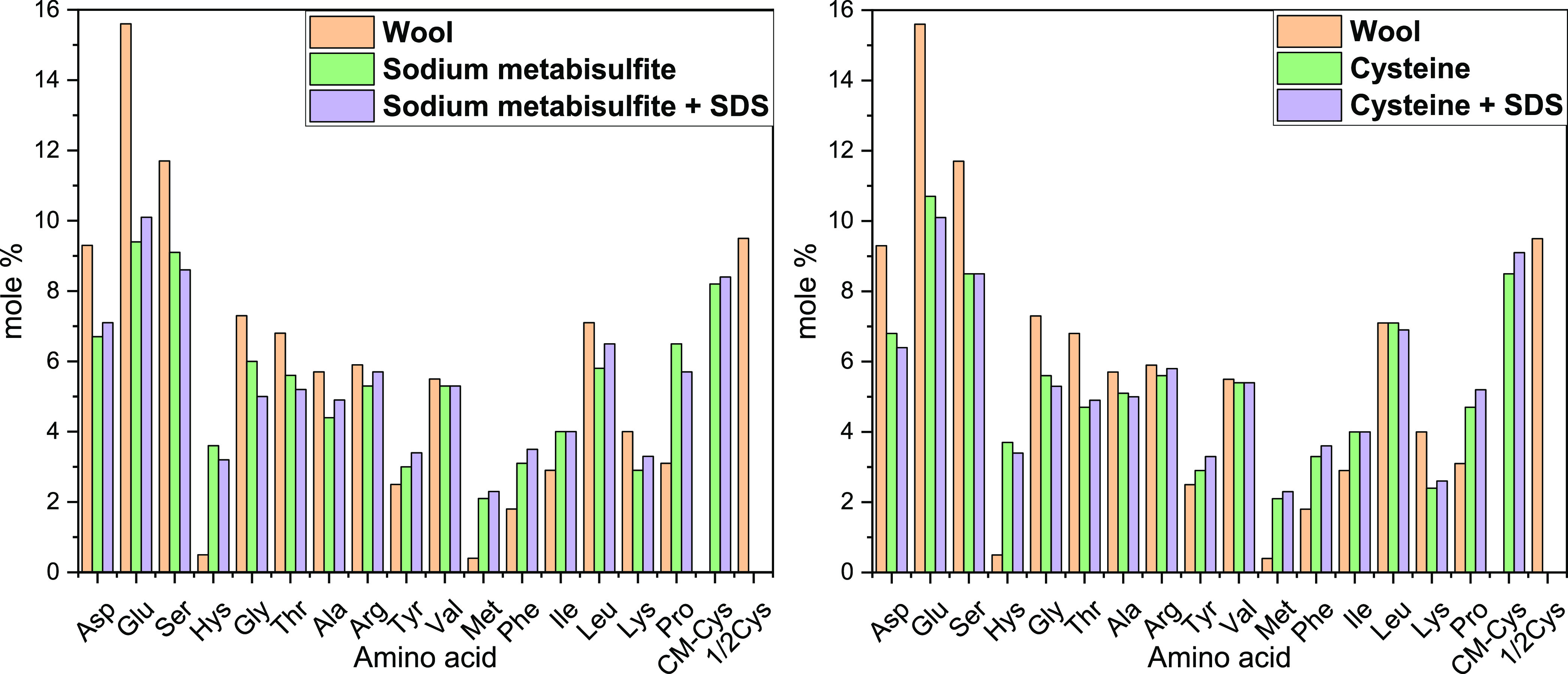
Amino acid composition (mol %) of samples extracted using sulfitolysis and reduction with cysteine compared to the original wool.^45^

The amino acid (AA) amount of the samples extracted using sulfitolysis and reduction with cysteine compared with the original wool^45^ is presented in Figure 2 and Table 1S. Hydrophilic amino acids include arginine, lysine, aspartic, and glutamic acids, asparagine, glutamine, serine, threonine, and histidine, while hydrophobic amino acids are glycine, proline, alanine, valine, isoleucine, leucine, methionine, tyrosine, phenylalanine, cysteine, and tryptophan.^46^ The preparative hydrolysis of keratin with HCl can cause various degrees of degradation in the AA residues, as well as their conversion or transformation to the other AA.^45,47^ All extracted samples show a similar AA profile. The acidic conditions completely destroyed tryptophan residues, similar to refs (26, 45). Moreover, similar to ref (26), the content of asparagine was considered as the sum of asparagine and aspartic acid, and the content of glutamine was calculated as the sum of glutamine and glutamic acid. The cysteine and cysteine residues in the wool fiber were detected as 1/2 cysteine.^46^ The extraction process slightly affected the cysteine content, most probably due to the disulfide bond cleavage at increased temperature and the release of sulfur as hydrogen sulfite, accompanied by the thiol group removal from the cysteine residues in the extracted keratin or other transformations.^47^ In addition, the cleavage of disulfide bonds can cause a considerable amount of peptides and free AA to be dissolved in water.^45^ Indeed, the most significant differences in the hydrolyzed samples can be observed in the amount of the hydrophilic amino acids (i.e., aspartic and glutamic acids, asparagine, glutamine, serine, threonine, or lysine), and these can be correlated with their solubility in water. Moreover, glutamic acid, aspartic acid, leucine, lysine, and arginine are the amino acids that contribute to the α-helix assembling of the low-sulfur proteins. However, cysteine, proline, serine, and threonine are the amino acids that constitute the high-sulfur proteins.^48^ In both cases, these proteins were slightly less abundant when compared to wool fibers (with the exclusion of proline). In general, most AA contents decreased, while few increased, including histidine, tyrosine, methionine, phenylalanine, isoleucine, and proline. These variances among the analyzed samples and wool from ref (45) might be ascribed to different source of wool or differences in the extraction efficiency that give diverse keratin populations that can be richer in certain amino acids.

**Figure 3:**
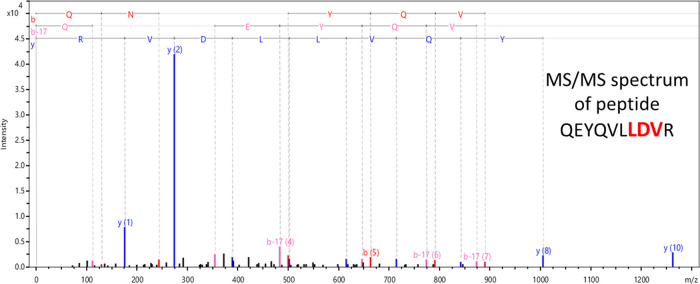
Representative tandem mass spectrum for the identification of QEYQVLLDVR peptide.

The keratin isoform (“genetic variations”^49^) was identified by MALDI-TOF/TOF MS/MS analysis performed on the keratins extracted from the gel electrophoresis bands in Figure 1c (lines 6 and 7, bands a–g). The results were confirmed by MS/MS and are summarized in Table 1. After in-gel digestion of selected bands, the keratin peptides were identified as sheep (*Ovis aries*) keratin when searched against SwissProt database using the Mascot search engine. Bands a–c correspond to keratin, type I microfibrillar 48 kDa, component 8C-1. In bands d–g of line 7, three keratin isoforms were identified: keratin type I microfibrillar 48 kDa, component 8C-1; keratin type II microfibrillar, component 7C; and keratin-associated protein 6-1. Since the motif LDV is of our interest, we specifically searched that motif in the tandem mass spectra. In tandem mass spectra, the peptides are fragmented and the mass difference between each peak corresponds to the molecular weight of the amino acid present in that peptide. Figure 3 shows a representative tandem mass spectrum for the identification of QEYQVLLDVR peptide containing the LDV motif.

**Table 1:** List of Peptides Identified per Each Sample

sample name	identified peptide at MS/MS leve	identified protein
a	QNQEYQVLLDVR	keratin, type I microfibrillar 48 kDa, component 8C-1 OS = *O. aries* OX = 9940 PE = 1 SV = 2
b	QNQEYQVLLDVR	keratin, type I microfibrillar 48 kDa, component 8C-1 OS = *O. aries* OX = 9940 PE = 1 SV = 2
	LNVEVDAAPTVDLNR	
	TVNALEVELQAQHNLR	
	AQYEALVETNRR	
c	QNQEYQVLLDVR	keratin, type I microfibrillar 48 kDa, component 8C-1 OS = *O. aries* OX = 9940 PE = 1 SV = 2
d	QNQEYQVLLDVR	keratin, type I microfibrillar 48 kDa, component 8C-1 OS = *O. aries* OX = 9940 PE = 1 SV = 2
	TVNALEVELQAQHNLR	
	AQYDDIASR	keratin, type II microfibrillar, component 7C OS = *O. aries* OX = 9940 PE = 1 SV = 1
	LEAAVTQAEQQGEVALNDAR	
e	AQYEALVETNRR	keratin, type I microfibrillar 48 kDa, component 8C-1 OS = *O. aries* OX = 9940 PE = 1 SV = 2
	QNQEYQVLLDVR	
	TVNALEVELQAQHNLR	
	LNVEVDAAPTVDLNR	
f	AQYEALVETNRR	keratin, type I microfibrillar 48 kDa, component 8C-1 OS = *O. aries* OX = 9940 PE = 1 SV = 2
	QNQEYQVLLDVR	
	TVNALEVELQAQHNLR	
g	SLCGSGYGYGSR	keratin-associated protein 6-1 OS = *O. aries* OX = 9940 GN = KRTAP6-1 PE = 1 SV = 2

**Figure 4:**
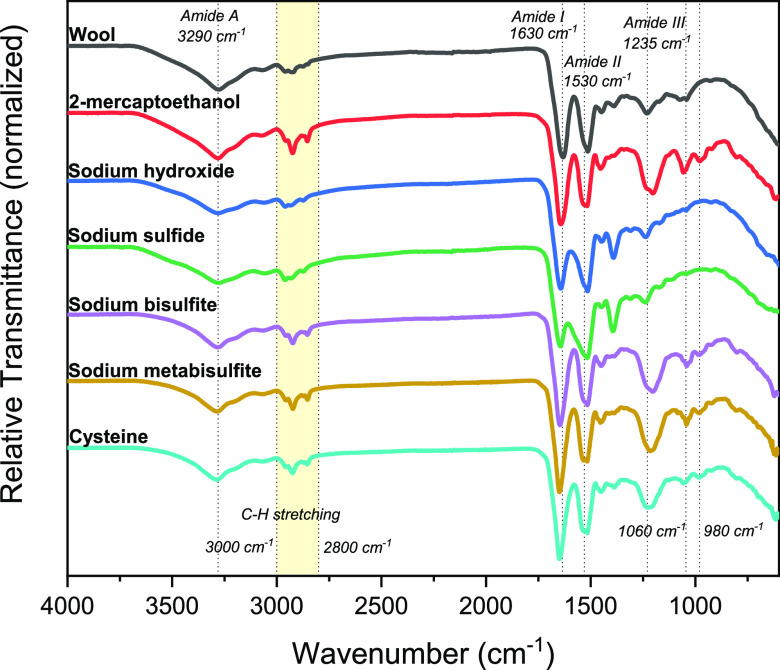
FTIR spectra of keratin extracted from wool using different extraction protocols. Highlighted bands are amide A, I, II, and III, Bunte salts, and the C–H stretching bonds region.

Fourier transform infrared spectroscopy (FTIR) was used to analyze the chemical composition of the extracted keratins and the influence of the extraction method on the chemical structure of the final product (Figure 4). The broad vibration band region between 3400 and 3250 cm^–1^ was attributed to the O–H and N–H stretching vibrations (Amide A). The bands that appeared in the range between 3000 and 2800 cm^–1^ were ascribed to C–H stretching bonds.^9,24,50^ Amide I was mainly associated with C=O stretching absorption in the range of 1700–1600 cm^–1^.^24^ Amide II was associated with N–H bending and C–H stretching vibration with absorption at 1540–1520 cm^–1^.^23,44^ While the bands between 1300 and 1220 cm^–1^ are ascribed to the amide III band due to the combination of N–H bending and C–N stretching vibration.^51,52^ The presence and position of these bands confirm the representative structure of the protein and indicate that the chemical structure of the proteins is retained after the extraction processes. In addition, the bands at 980 and 1060 cm^–1^ can be related to asymmetric and symmetric S=O stretching vibrations (cysteine-*S*-sulfonated residues, Bunte salts), respectively.^9,10,24^

This screening of extraction protocols was performed because, despite the fact that numerous papers describe the different methods used here with several variations, some inconsistencies are present. For instance, the yield was calculated differently (i.e., spectrophotometrically^41^ or via Bradford method^53^), and the molecular weight of the product in some studies was not reported. Therefore, this report provides an extensive study of the versatile keratin extraction methods that are available in the literature. It complements the analyses that are crucial from industrial point of view, completing missing information; consequently, it can become an initial position for the keratin-based materials development.

As confirmed by FTIR, all selected extraction protocols preserved the overall chemical structure of the proteins. The keratins prepared with sulfitolysis and reduction with cysteine showed a good compromise between the molecular weights (both protocols produced keratins in the range of 10–60 kDa) and yield (63 and 56%, respectively); moreover, these methods are based on nontoxic reagents. Therefore, these two methods were chosen for the preparation of keratin particles for cell guidance applications.

### 3.2 Isoelectric Precipitation

**Figure 5:**
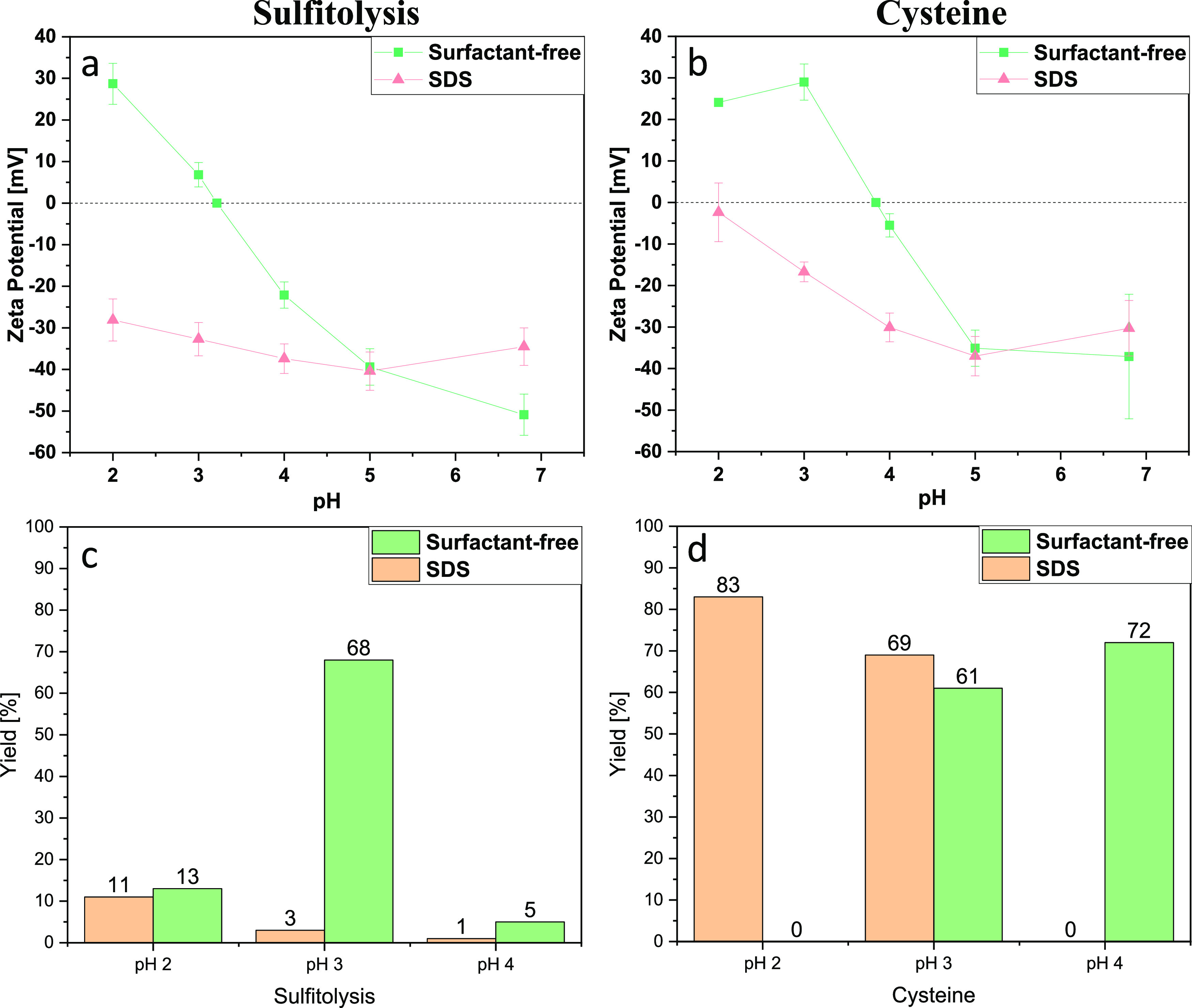
(a, b) Zeta potential (mV) of keratin dispersions prepared using sulfitolysis (a) and cysteine reduction (b) at different pH values. (c, d) Precipitation yield of dried keratins prepared using sulfitolysis (c) and cysteine reduction (d) at different pH values.

**Scheme 1:**
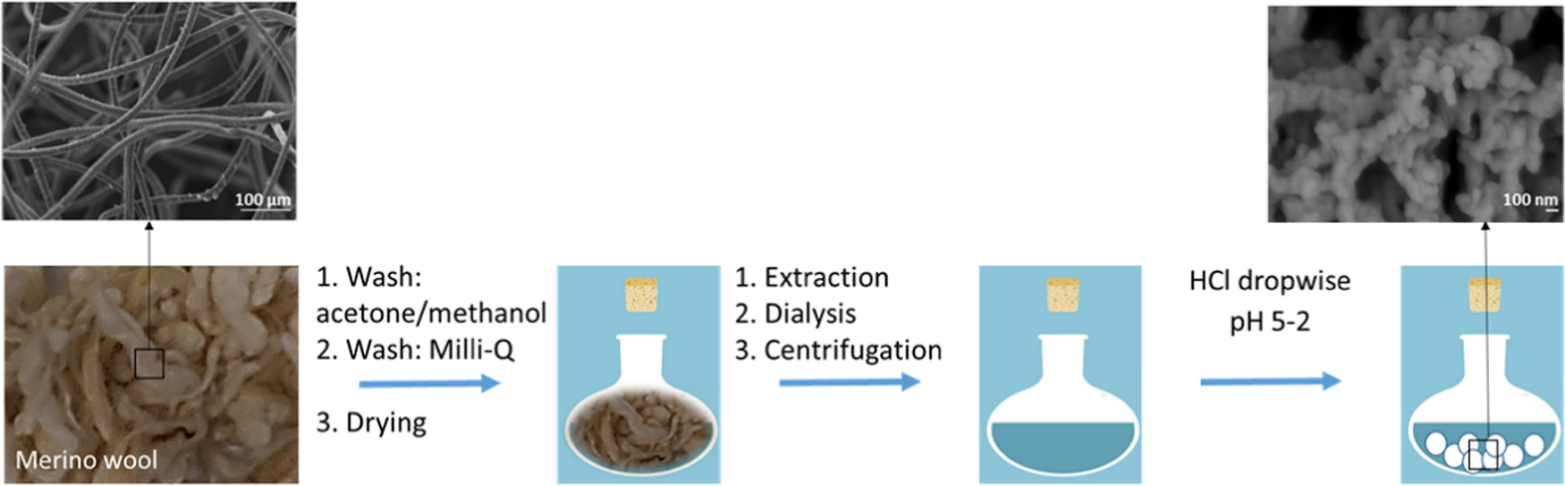
Schematic Illustrating the Precipitation Protocol of Keratin Particles

Keratin particles were prepared by isoelectric precipitation from keratin water solutions (Scheme 1). To identify the isoelectric point, the pH of the keratin solutions was decreased from 7 to 2, and the zeta potential was measured at each pH value (Figure 5a,b). All samples demonstrated a strong net surface charge at high or low pH values and evident sigmoidal change from negative to positive with decreasing pH and a neutral point that indicated the isoelectric point. The sample prepared by reduction with cysteine showed an isoelectric point at around pH 4, comparable with keratins originating from wool.^4,15^ However, the sample obtained via the sulfitolysis process exhibited an isoelectric point at pH 3. This shift to lower pH can be explained by the formation of negatively charged sulfonate groups created by the sulfitolysis of cysteine. The samples prepared in the presence of SDS showed negative zeta potential values within the whole pH range; this could be due to the remaining traces of SDS after dialysis and the formation of the complex between keratin and surfactant. A similar trend has been observed for the soy protein isolate, where the charge of the SDS-containing samples was high and negative at all pH values, while surfactant-free samples showed sigmoidal changes from positive to negative with increasing pH.^54^

When the pH of the keratin solutions is close to the isoelectric point, the proteins precipitate into particles due to the lack of ionic repulsion between them. The yield of the precipitate collected at different pH values is shown in Figure 5c,d. The highest precipitation rate of keratins achieved via the sulfitolysis process was 68% for the surfactant-free sample and 11% for the sample containing SDS. The precipitation efficiency of keratins obtained via reduction with cysteine was 72% for the sample extracted without surfactant and 83% for the sample extracted in the presence of SDS. These results are consistent with the zeta potential evaluation. The yield of the samples prepared without surfactant is the highest at their isoelectric point, while the yield of the samples prepared in the presence of surfactant is the highest at pH 2, where the zeta potential values are the closest to 0 mV.

**Figure 6:**
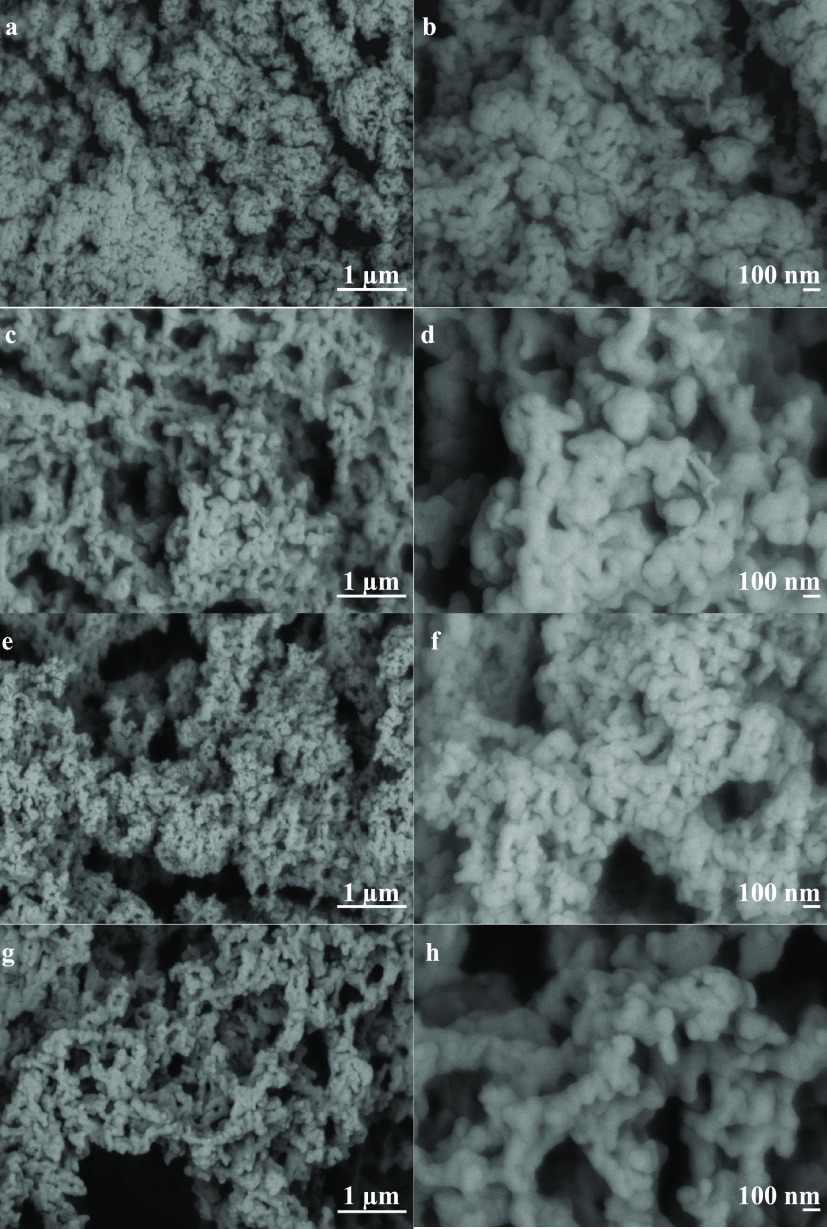
SEM images of keratin precipitates prepared using isoelectric precipitation and two extraction protocols—reduction with cysteine (a, b—KCPs; c, d—KCPs + SDS) and sulfitolysis (e, f—KSPs; g, h—KSPs + SDS). The samples were collected at the pH that gave the highest yield.

The surface morphology of the precipitated keratins at the pH that gave the highest yield was analyzed by SEM. The isoelectric precipitation process leads to globular, tightly packed nano- and microparticles with the size of ∼60–100 nm for the surfactant-free samples, ∼80–150 nm for the samples prepared with SDS, randomly arranged structures and agglomerates with the size of ∼0.8–1.3 μm for the surfactant-free samples, and ∼1.0–1.5 μm for the samples prepared with SDS (Figure 6). The size of the agglomerates is in agreement with DLS analysis. The hierarchical morphology, with small nanosized particles clustered together in microsized aggregates, is similar to the keratin particles synthesized in previous studies.^24,25^ It can be noted that the presence of SDS during the extraction influenced the morphology obtained after the isoelectric precipitation, with particles characterized by a smoother surface; this was probably due to the complex formation between the surfactant and the keratin molecules that prevents the protein chains from aggregation.^43^

**Figure 7:**
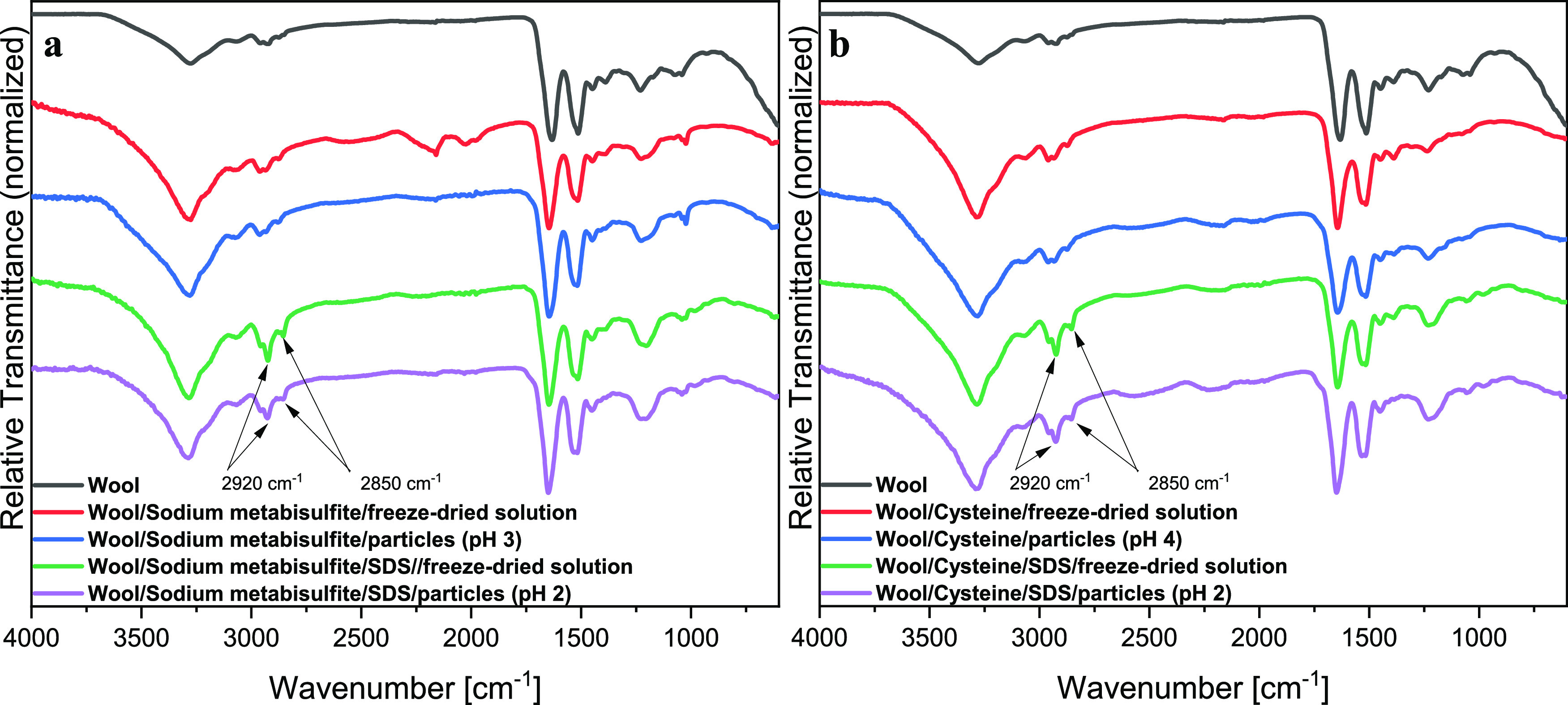
FTIR spectra of wool, keratin extracted from wool using (a) sodium metabisulfite and (b) cysteine treatment, and particles resulting from isoelectric precipitation. Highlighted bands at 2920 and 2850 cm^–1^ can be ascribed to the C–H vibration of alkanes present in the SDS-containing samples.

Figure 7 reports the FTIR spectra of wool, freeze-dried keratin prepared via sulfitolysis and reduction with cysteine, and their precipitated analogues. Apart from the characteristic absorption bands of proteins, the samples prepared with SDS show additional bands at 2920 and 2850 cm^–1^ that can be ascribed to the C–H vibration of alkanes;^20^ further evidence is the absorbance peak at 1220 cm^–1^ that can be ascribed to skeletal vibration involving the bridge S–O stretch,^55^ confirming the presence of SDS–keratin complexes that cause the increased stability of keratin in solution, and hence the higher extraction yield and the differences seen in the zeta potential of keratins in solution.

While the freeze-dried keratin solutions right after extraction resulted in a water–soluble powder, keratin particles obtained by isoelectric precipitation were water–insoluble, indicating that the disulfide bridges (intramolecules and intermolecules) were reformed. Keratin particles present superior water stability, providing an advantage over other protein materials such as albumins.^31,56^

The cytotoxicities of KCPs, KCPs + SDS, KSPs, and KSPs + SDS were studied using primary human dermal fibroblasts (HDFa cells) as a model cellular system. Cell viability results via MTS assay on HDFa cells grown in the presence of extraction media obtained after incubating 1 mg/mL of KCPs and KSPs in a cell medium for 24 and 48 h are reported in Figure 6S. The particle-free media was used as the control. For this type of assay, various particles under study were incubated in a cell culture medium and the extract was used to assess their indirect cytotoxicity. As evident from the results presented in Figure 6S, residues of the samples prepared without surfactant showed good cytocompatibility, with a slightly increasing trend between 24 and 48 h for the KCPs. On the contrary, the particles obtained from keratin extracts containing residues of SDS were cytotoxic in both cases, showing a significant decrease in cell viability percentage compared to control (*p* < 0.01); therefore, only samples KCPs and KSPs were considered for the cell guidance experiments.

Similar to keratin particles from ref (20), keratin particles obtained via isoelectric precipitation can be loaded with different molecules to induce their bioactivity. To evaluate the encapsulation efficiency, keratin particles were prepared from the keratin solutions where the model drug (methylene blue or FITC-albumin) was dissolved. The specific color of the particles (yellow for FITC-albumin and blue for methylene blue) suggests successful drug encapsulation as presented in Figure 2S. Moreover, the encapsulation efficiency was studied spectrophotometrically (Figure 3S). Interestingly, when comparing the two absorption peaks of FITC-albumin with the absorption peak of supernatant, the absorption after encapsulation dropped drastically and reached nearly 0, suggesting that the drug was encapsulated in KSPs and KCPs. However, the encapsulation efficiency of methylene blue was poorer, particularly for KCPs, since the absorption peaks before and after encapsulation are almost identical. In contrast, the absorption peak of the supernatant after KSP encapsulation decreased twice, suggesting higher encapsulation efficiency.

To sum up, our proposed simple and effortless methods led to the fabrication of particles characterized by high yield, good surface morphology, maintained protein structure, water insolubility, cytocompatibility, and loading drug capability, thus making this approach an attractive and cost-effective alternative to the current methods, which rely on multiple experimental steps or advanced equipment.

### 3.3 Keratin Particle Micropatterns

**Figure 8:**
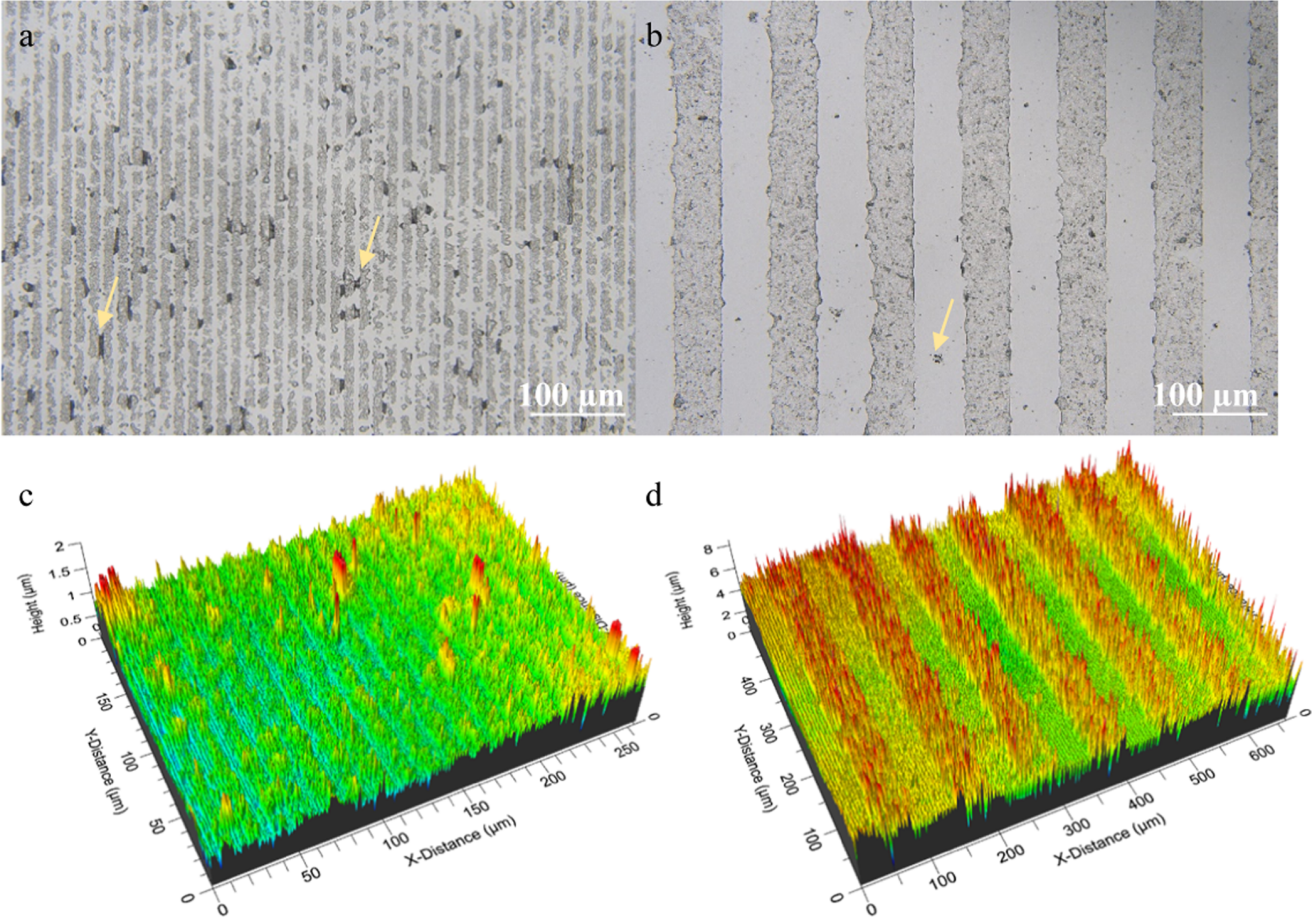
Optical profilometer 2D images of (a) 5–10 KCPs and (b) 50–50 KCPs and 3D rendering of (c) 5–10 KCPs and (d) 50–50 KCPs. Yellow arrows indicate the presence of KPs between the stripes.

The prepared cytocompatible keratin particles were used as a keratin-based ink for the microcontact printing (MCP) process with a patterned PDMS stamp. The patterns were obtained by (1) inking the hydrophilized PDMS stamps with the suspension of keratin particles, (2) drying, and (3) placing the stamp in contact with a flat surface, such as a glass coverslip. Several factors are crucial in the process, such as the ink concentration, the type of dispersant, and the time of each step, even more so when working with a discrete, microparticulate system. Therefore, these parameters were taken into account to optimize the MCP process. Pristine PDMS is strongly hydrophobic, while effective printing requires a well-defined wettable surface; therefore, PDMS stamps were plasma oxidized, acquiring hydrophilicity and enabling superior dispersion of the particles on the PDMS surface. The dispersion medium was then dried with an inert gas; however, contrary to the standard MCP, the affinity of the particles to the PDMS stamp was higher than their affinity to the glass resulting in a poor transfer of the particles onto the glass surface. This is most probably due to the strong affinity of the various hydrophilic groups present on the surface of keratin particles to the hydroxyl groups on the oxidized PDMS stamps. The driving forces involved in transferring keratin to the glass are likely based on van der Waals, hydrophobic, solvation, electrostatic, and capillary interactions.^57^ The balance of the adsorption energies is essential for a successful pattern transferring; the particles should adhere to the oxidized PDMS stamps but should disengage in favor of a stronger interaction with the chosen substrate. Significant improvement of the transfer of keratin particles onto the glass surface was achieved by wetting with 15 μL of ethanol and subsequent incomplete drying with N2 flow (leaving a layer of ethanol on the surface of the stamp) that induced affinity of the particles to the glass and, ultimately, improved their printability. Our printing process was inspired by the printing of protein solution Semaphorin 3A (where the detailed protocol described the PDMS microfabrication and substrate micropatterning was presented)^36^ and the printing of Tobacco mosaic virus particles.^37^ In Figure 4S, optical profilometer images of the two different patterns obtained with the two keratin particles under study are reported. To the best to our knowledge, this is the first report describing the microcontact printing of keratin protein particles. The patterns appear nodular due to the size of the printed keratin particles. As can be seen from the DLS data reported in Figure 5S, the keratin particle size in the ink that was used for printing was around 1000 nm. In general, 50–50 patterns more precisely reflect the PDMS stamps when compared to the 5–10 patterns (Figures 8 and 4S). The higher number of defects that can be seen in the smaller patterns can be due to the fact that a 5 μm wide space between two neighboring stripes is insufficient for a precise accommodation of the micron-sized particle aggregates, resulting in a less defined topography. On the other hand, KCP-based 50–50 patterns show the most uniform stripe morphology (Figure 4Sa,b). Among all dispersants tested, ethanol led to the fabrication of the most uniform patterns.

### 3.4 Assessment of Cell Morphology and Guidance

**Figure 9:**
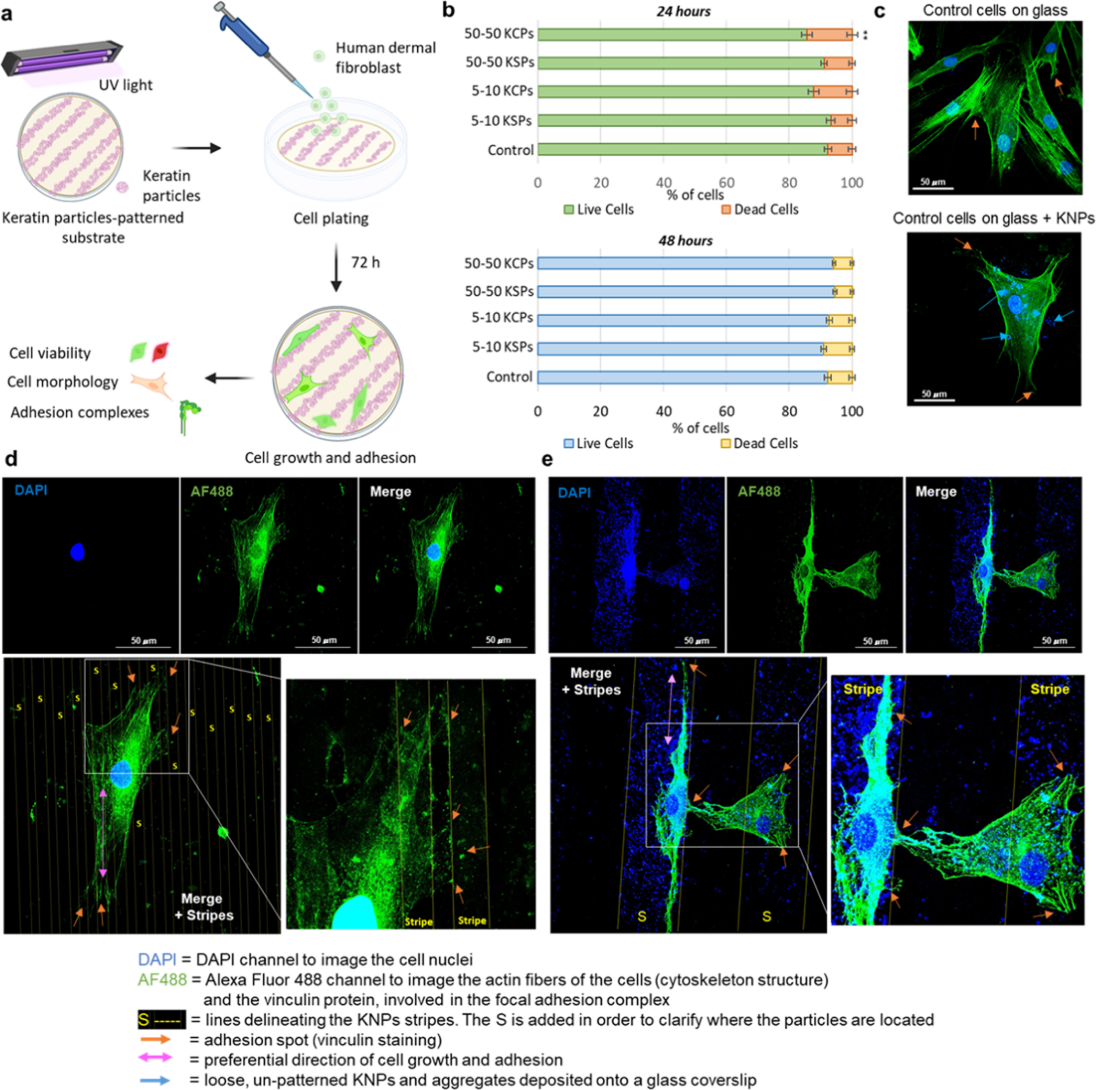
(a) Schematic representation of the cell plating and analysis procedure involving the KP-based micropatterned substrates. (b) Results of the live/dead staining experiments, where HDFa cells were let growing in contact with the different KP-based micropatterned substrates under study for 24 and 48 h. A Student’s *t*-test was performed considering *p* < 0.01 (**). (c) Confocal images of HDFa cells in the control experiment: upper image—cells plated and grown for 72 h onto a plain glass coverslip; lower image—cells plated and grown for 72 h onto a glass coverslip covered coated with 1 mg/mL of KCPs (unpatterned). Blue arrows indicate loose, unpatterned KCPs deposited onto the glass surface. Orange arrows indicate the presence of focal adhesion complexes as a result of vinculin staining. (d, e) HDFa cells grown for 72 h in contact with 5–10 KCP and 50–50 KCP stripes, respectively. The stripes were visualized by leveraging on the KP auto-fluorescence signal in the blue channel, and then their contour was overlaid onto the merged image. The locations of the stripes are indicated by the letter “S”. Construction of the overlay for the images in panels (d) and (e) can be found in the Supporting Information (Figures 8S–9S). The DAPI (blue) channel highlights the nuclei of the cells and, thanks to their auto-fluorescence signal, the KPs as well. The AF 488 (green) channel was used to image both the cytoskeleton and the focal adhesion complex of the cells. Orange arrows indicate the presence of focal adhesion complexes as a result of vinculin staining. Pink arrows indicate the preferential direction of growth for the stretched, adherent cells. Scale bar 50 μm.

In this study, HDFa cells were directly plated on two different types of patterns (5–10 and 50–50) made out of two different types of particles (KCPs and KSPs) to evaluate their ability in cellular guidance in vitro (Figure 9a). After 72 h of growing and adhesion, the samples were tested for cell viability, morphology, adhesion, and arrangement from the primary pattern-cell interactions.

To assess the cytocompatibility of the keratin-based micropatterns, live/dead staining was performed with the cells directly plated onto the patterned samples. In general, good cytocompatibility is required for medical applications and the cell viability should be >80%.^58^ Following the ISO10993-5:2009 norm, HDFa cells were let grow in contact with the different KP-based micropatterned substrates for 24 and 48 h to evaluate their direct cytotoxicity. Cell viability obtained from live/dead assays was above 80% for all of the selected samples (Figure 9b) at both time points considered. However, after 24 h, the cell viability of the 50–50 KCP pattern was significantly different compared to the control (*p* < 0.01), showing a decrease in the live cell number. Nonetheless, this difference was recovered after 48 h of cell growth. This result further supports the cytocompatibility previously observed with the MTS assay for the fibroblasts grown in the presence of extraction media obtained after the incubation of 1 mg/mL of KCPs and KSPs in a cell medium (Figure 6S), corroborating the choice of these microparticulate materials for cell culture applications.

Figures 9d–e and 7S–11S report the confocal imaging study of the HDFa cells grown for 72 h on the keratin particle-based patterns. The stripes created a favorable environment for cell spreading and attachment probably because of the high surface area of the hierarchical keratin particles and the presence of the LDV adhesion sequences on the particle surfaces. In tissue engineering, complex substitutes are needed to mimic cell alignment, architecture, and function of the tissue that we want to restore/regenerate. Cell adhesion to a surface in vitro comprises three different stages, such as sedimentation (phase I), cell attachment (phase II), and cell spreading and stable adhesion (phase III).^59^ One of the main factors involved in the last phase is the formation of focal adhesion spots. Cell adhesion to a biomaterial or a scaffold can be characterized via the investigation of vinculin, which plays a crucial role in the integrin-mediated adhesion by acting as a link between the cytoskeleton (i.e., actin fibers arrangement and tension) and the extracellular environment.^60^ Vinculin is involved not only in the adaptation of tissues to forces (it transmits forces from inside the cell to the extracellular matrix, subsequently regulating the cellular response to mechanical stimuli) but also plays an important role in determining focal adhesion stability.^60,61^ The precise positioning of vinculin and the 3D organization of the focal adhesions play a crucial role in the stability of formed focal adhesion, their ability to sustain mechanical forces and transmit downstream signals.^60,61^ When vinculin is activated, focal adhesions are formed and are able to mature, thus allowing the cells to sense and interact with the surrounding.^61^ As a result, vinculin visualization is often used for the identification of the focal adhesion spots pertaining to a cell plated onto a specific substrate.^59^ Different strategies can be implemented to provide instructive environments for cellular growth and orientation, including topographically and chemically functionalized surfaces.^61,62^ The results presented in Figure 9 clearly show how the cells are following the direction of the printed patterns and preferentially attaching to the patterned surface, as highlighted by the orange arrows pointing to the focal adhesion points (vinculin staining). Figures 9d–e and 7S–11S represent the HDFa cells grown in contact with the KCP-based patterns (5–10 and 50–50). In the case of the 5–10 KCP stripes (Figure 9d), the cell of micrometric dimensions (length > 150 μm and width around 50 μm) appears spreading along multiple keratin stripes; however, the preferential direction of cell growth is parallel to the particle stripe and the focal adhesion complexes are highly colocalizing with the pattern (orange arrows in Figure 9d zoomed inset, Figures 7S–8S). A similar preferential growth was observed for the case of the 50–50 KCP-based pattern, where, given the dimensions of this topographical arrangement that are more comparable to the fibroblast length and width proportions, a single cell preferably lays along one stripe (Figures 9e and 9S). This behavior was likewise noticed for cells plated onto the 5–10 and 50–50 KSP-based patterns (Figures 10S and 11S) where pink double-ended arrows indicate the direction of the stripes. Despite the fact that the 5–10 KSP patters appeared less precise, with a higher presence of particle aggregates, vinculin staining highlighted once again the colocalization of the adhesion complexes with the particle aggregate borders (Figure 11S, zoomed insets), confirming that in all studied cells, the focal adhesion complexes were colocalized with the keratin particles.

Interestingly, the confocal imagining study of KSP-based 50–50 patterns (Figure 11S) highlights the ability of the cells to form membrane protrusions and to favor adhesion via the generation of focal adhesion points localized at the KSPs–cell interface. This indicates that our microparticulate keratin stripe design constitutes a suitable environment for fibroblasts to (1) recognize the pattern, (2) influence their adhesion, and (3) instruct a preferential growth direction. To the best of our knowledge, this is the first time this has been shown on the patterns of keratin particles.

As a control experiment to double-check our hypothesis and assess the overall cell morphology, unpatterned samples were prepared by depositing drops of keratin particle suspensions at a concentration of 1 mg/mL onto glass coverslips. Figures 9c and 6Sb,c represent the resulting confocal imaging investigation of the control experiment, where the blue arrows indicate loose, randomly deposited particles. The cells seeded on both controls prepared via random deposition of KCPs and KSPs revealed a spread-out morphology with no specific directionality of adhesion/growth, characterized by well-defined and stretched actin filaments, indicative of healthy fibroblasts, comparable with the control cells plated on uncoated glass coverslips (Figure 6Sa). The focal adhesion points appear randomly distributed around the cell membrane borders. This experiment further confirmed that the presence of the keratin patterns did affect the way the fibroblasts respond and adhere to the substrate.

As an advantage over synthetic materials (e.g., poly(methyl methacrylate), PDMS, polystyrene, polyimide) usually applied for cellular growth and guidance applications, that do not contain any biofunctional groups easily recognizable by the cell membrane molecules, keratin can leverage on the LDV motifs in its polypeptide structure that is a target for integrin binding.^63,64^ This study suggests that patterning a substrate with keratin, thanks to the combination of topography with the bioactivity of keratins, constitutes a promising strategy to provide biochemical cues for fibroblast adhesion and even ease their alignment. This programming of surfaces could have potential applications in tissue engineering, where facilitating adhesion and directing cell growth can have potential benefits for cell colonization of scaffolds.

## 4 Conclusions

Keratin was extracted from wool using various treatments adapted from the literature. The most effective methods were sulfitolysis and reduction with cysteine, that were selected for further experiments. The isoelectric point of keratin prepared using sulfitolysis was at pH 3, while the sample obtained by reduction with cysteine showed an isoelectric point at pH 4. Keratin particles could be rapidly synthesized by precipitation at their respective isoelectric points, leading to a high yield production (68 and 83% for keratins from sulfitolysis and cysteine, respectively). The morphology of the keratin particles was in the form of globular, tightly packed nano- and microparticles (with the size between ∼60 and 150 nm) and randomly arranged structures and aggregates (with the size between ∼0.8 and 1.5 μm). For the first time, a microcontact printing strategy for keratin particles was proposed, developing an appropriate ink by optimizing the solvent used for dispersing keratin particles to achieve good dispersion and appropriate interaction with PDMS mold. Two different keratin micropatterns were prepared (5–10 and 50–50 μm) using two different types of keratin particles, KSPs, and KCPs. The keratin particles demonstrated cytocompatibility toward primary human adult fibroblasts, both in direct contact and as the medium extract, and created a suitable environment for cell attachment and spreading. Moreover, the presence of LDV motifs, a tripeptide promoting cell adhesion, on the extracted keratin was confirmed by the MALDI-TOF/TOF MS/MS analysis. For the first time, we demonstrated that this LDV could be leveraged by patterning surfaces with keratin particles. The presence of keratin micropatterns not only facilitated HDFa cell adhesion but also appeared to steer their overall orientation and elongation, thus envisaging a potential to program surfaces to improve cell growth and to provide biochemical cues to direct cell growth in tissue engineering applications. To conclude, owing to its inherent cytocompatibility, its biological functionalities, and deriving from a renewable resource, our proposed protein particle-based micropatterns represent for the first time a promising alternative to the synthetic materials currently employed as tissue regeneration templates.

## Supporting Information Available

Images of keratin particles loaded with the drug, UV–vis, table with amino acid analysis, SEM images of silicon-based master molds, optical profilometer images of KCP and KSP patterns, dynamic light scattering of KCPs and KSPs, cell viability results via the MTS assay on HDFa cells, confocal images of the HDFa cells cultured in the control experiments, and HDFa cells onto 5–10 and 50–50 KCP or KSP stripes (PDF)

## Abbreviations

MALDI-TOF/TOF MS/MS: matrix-assisted laser desorption/ionization coupled with time-of-flight tandem mass spectrometer analysis
LDV: leucine–aspartic acid–valine
SDS: sodium dodecyl sulfate
IFPs: intermediate-filament proteins
KAPs: keratin-associated proteins
MCP: microcontact printing
PDMS: polydimethylsiloxane
MWCO: molecular weight cut-off
SDS-PAGE: sodium dodecyl sulfate-polyacrylamide gel electrophoresis
DTT: dithiothreitol
OPA: orthophthalaldelyde
FMOC: 9-fluorenyl-methyl-chloroformate
MeCN: acetonitrile
TFA: trifluoroacetic acid
HCCA: α-cyano-4-hydroxycinnamic acid
KSPs + SDS: particles prepared starting from the keratin solution extracted via sulfitolysis in the presence of SDS
KSPs: particles prepared starting from the keratin solution extracted via sulfitolysis
KCPs + SDS: particles prepared starting from the keratin solution obtained by reduction with cysteine in the presence of SDS
KCPs: particles prepared starting from the keratin solution obtained by reduction with cysteine
FTIR: Fourier transform infrared
ATR: attenuated total reflectance
SEM: scanning electron microscopy
HDFa: primary adult human dermal fibroblast
PBS: phosphate buffer saline
DAPI: 4′,6-diamidino-2-phenylindole, dihydrochloride
MW: molecular weight
AA: amino acid
