# Supplementary material for: Wool Keratin Nanoparticle-Based Micropatterns for Cellular Guidance Applications
Source: ACS Appl Nano Mater. 2022 Oct 4;5(10):15272–87. doi: 10.1021/acsanm.2c03116 (PMC9624257; doi:10.1021/acsanm.2c03116)
Supplement: Supplementary file 1 — an2c03116_si_001.pdf [file an2c03116_si_001.pdf]

Supporting Information for:

Wool Keratin Nanoparticle-Based Micropatterns For Cellular Guidance Applications.

Dagmara J. Trojanowska[1,2], Giulia Suarato[1,3,§], Clarissa Braccia[4], Andrea Armirotti[4], Fabrizio Fiorentini[1], Athanassia Athanassiou[1], Giovanni Perotto[1]*

[1]Istituto Italiano di Tecnologia, Smart Materials Group, Via Morego, 30, 16163 Genova (Italy),

[2]Department of Materials Science, University of Milano-Bicocca, via R. Cozzi 55, 20125 Milan (Italy).

[3]Istituto Italiano di Tecnologia, Translational Pharmacology Facility, Via Morego, 30, 16163 Genova (Italy).

[4]Istituto Italiano di Tecnologia, Analytical Chemistry Facility, Via Morego, 30, 16163 Genova (Italy).

* Giovanni Perotto – Istituto Italiano di Tecnologia, Smart Materials Group, Via Morego, 30, 16163 Genova (Italy), Email: Giovanni.Perotto@iit.it

Amino acid	Wool (mol%) ([67])	Cysteine (mol%)	Cysteine + SDS (mol%)	Sodium metabisulfite (mol%)	Sodium metabisulfite + SDS (mol%)
Asp	9,3	6,8	6,4	6,7	7,1
Glu	15,6	10,7	10,1	9,4	10,1
Ser	11,7	8,5	8,5	9,1	8,6
Hys	0,5	3,7	3,4	3,6	3,2
Gly	7,3	5,6	5,3	6,0	5,0
Thr	6,8	4,7	4,9	5,6	5,2
Ala	5,7	5,1	5,0	4,4	4,9
Arg	5,9	5,6	5,8	5,3	5,7
Tyr	2,5	2,9	3,3	3,0	3,4
Val	5,5	5,4	5,4	5,3	5,3
Met	0,4	2,1	2,3	2,1	2,3
Phe	1,8	3,3	3,6	3,1	3,5
Ile	2,9	4,0	4,0	4,0	4,0
Leu	7,1	7,1	6,9	5,8	6,5
Lys	4	2,4	2,6	2,9	3,3
Pro	3,1	4,7	5,2	6,5	5,7
CM-Cys		8,5	9,1	8,2	8,4
1/2Cys	9,5				

Table 1S. Amino acid composition (mol%) of samples compared to original wool [65].

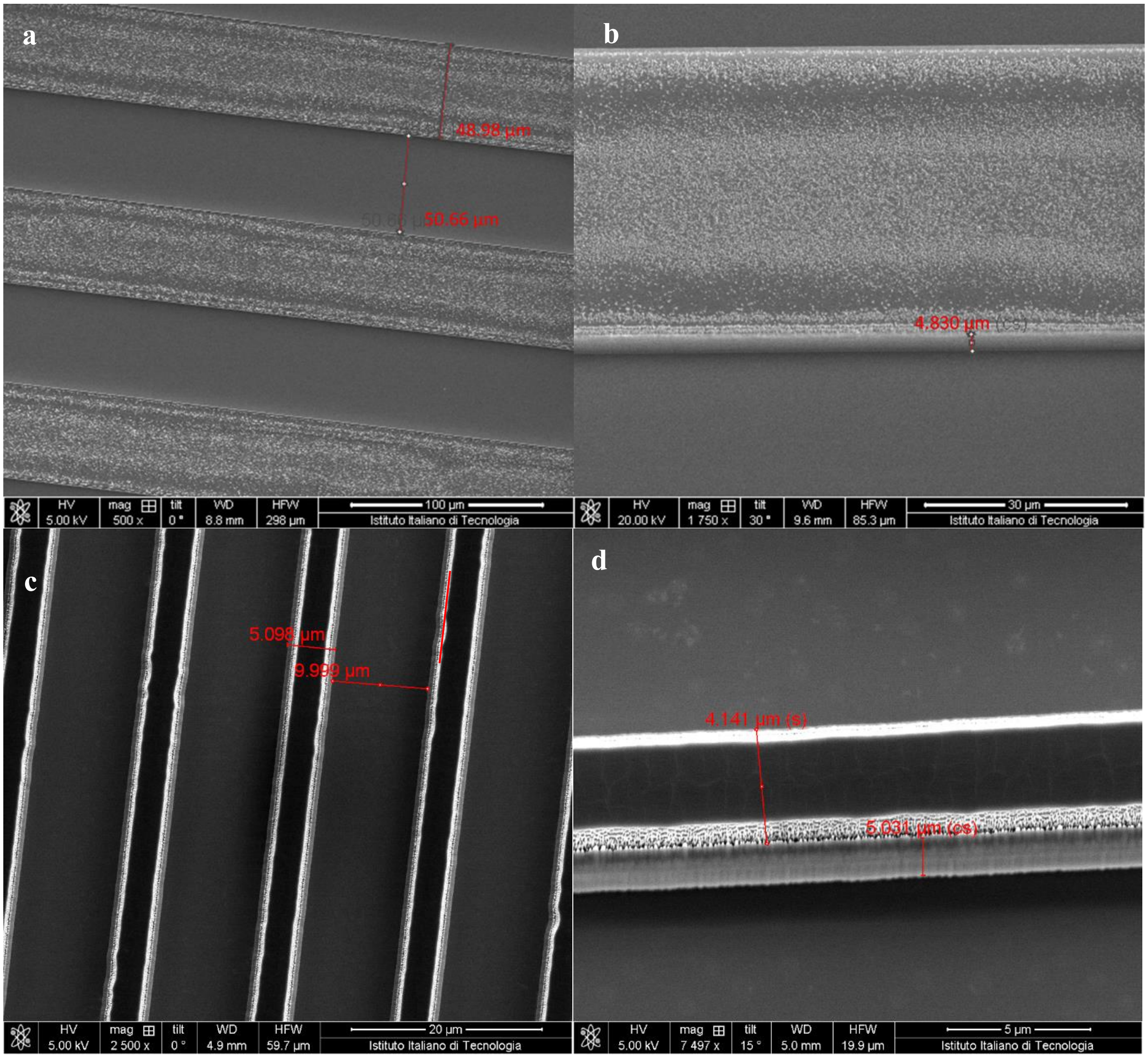

Figure 1S. SEM images of the silicon-based master molds a-b) 50 -50 pattern, c-d) 5-10 pattern.

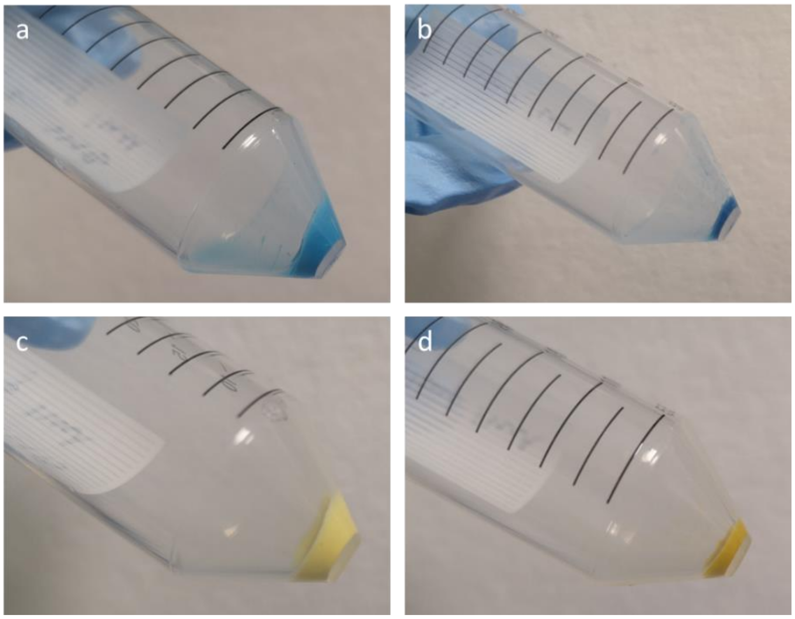

Figure 2S. Keratin particles. a) KCPs loaded with methylene blue; b) KSPs loaded with methylene blue; c) KCPs loaded with albumin-FITC; d) KSPs loaded with albumin-FITC.

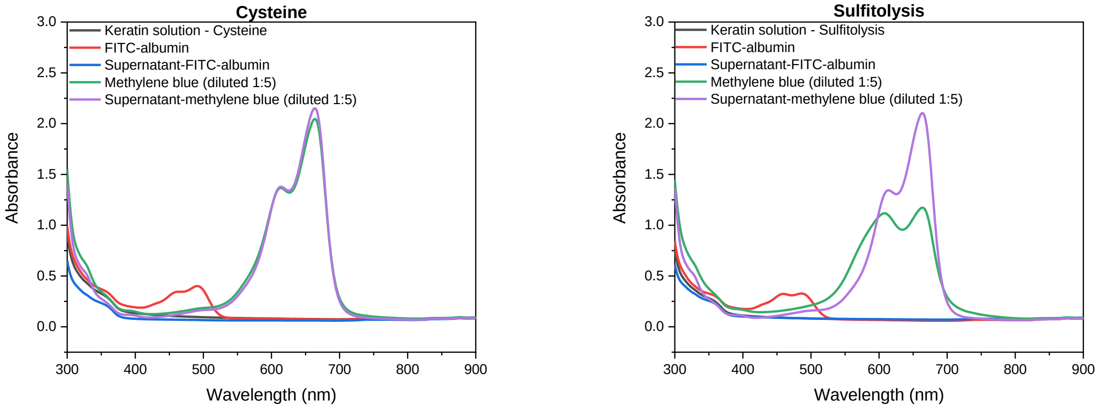

Figure 3S. UV-VIS spectra of keratin solutions a) reduction with cysteine, b) sulfitolysis; model drugs (FITC-albumin, methylene blue) and supernatant from the drug ecapsulation.

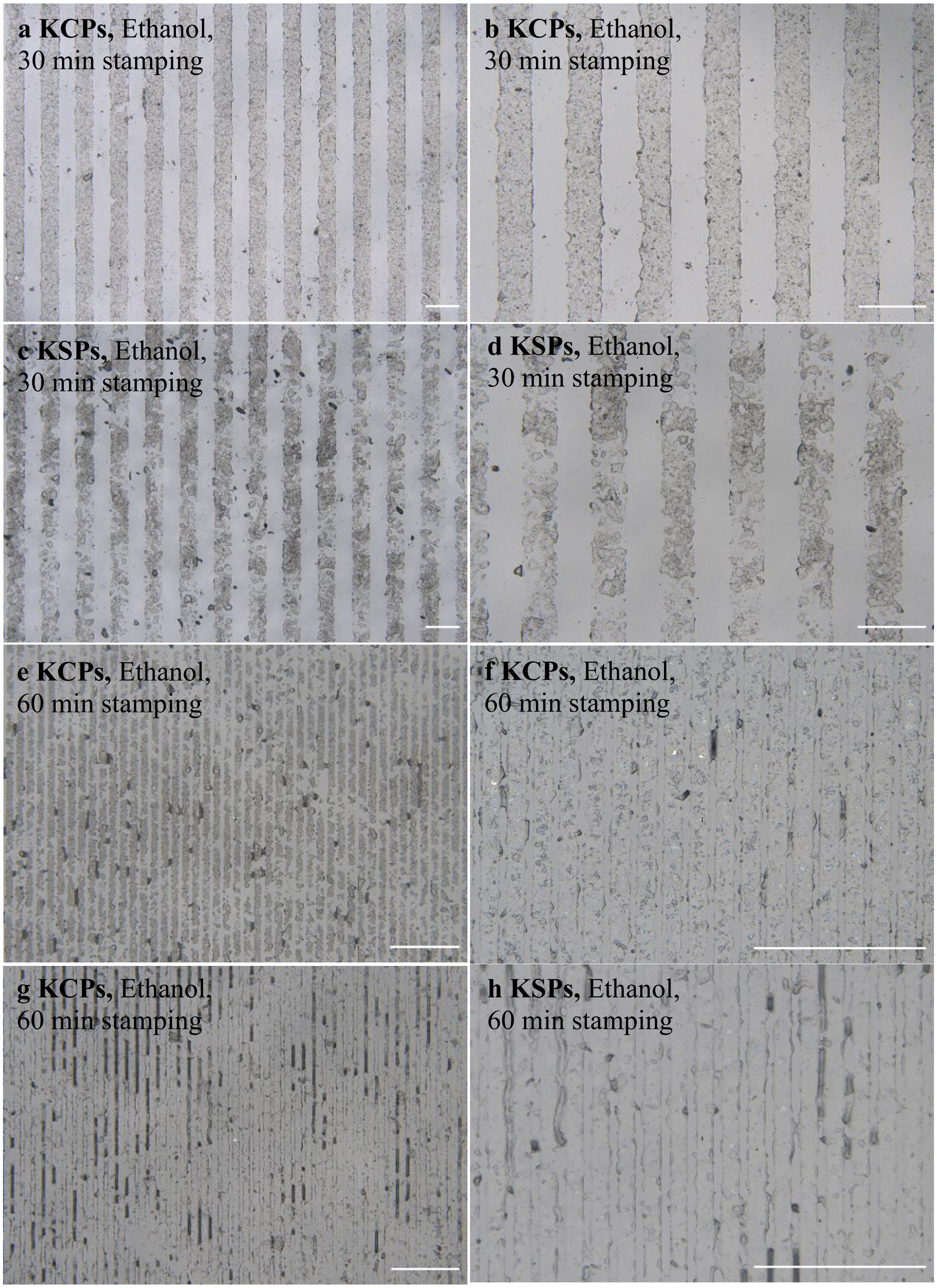

Figure 4S. Selected optical profilometer images of KCPs and KSPs 50 μm – 50 μm patterns and their magnifications (a-d) and the corresponding 5 μm – 10 μm patterns and their magnifications (e-h). Scale bar 100 μm.

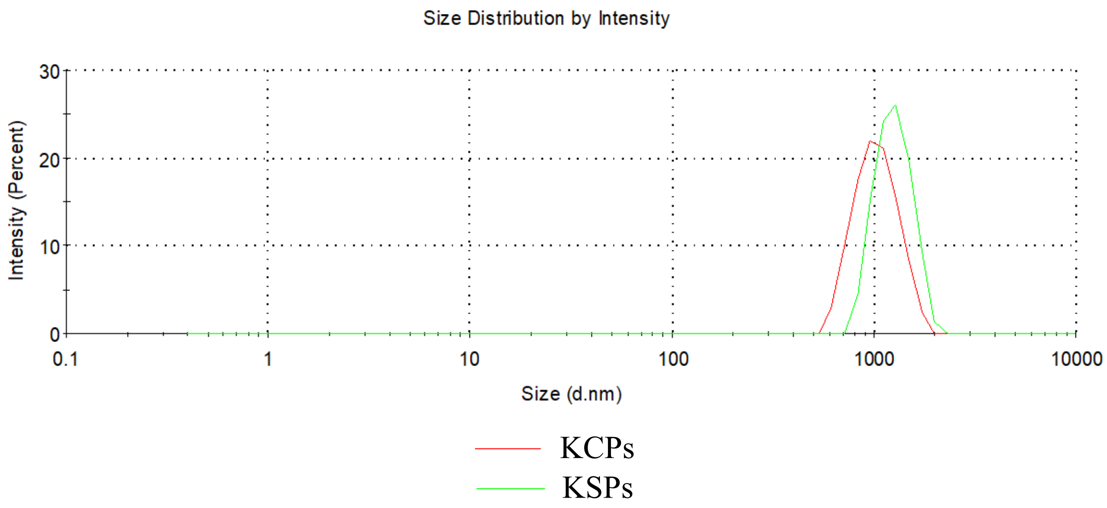

Figure 5S. Dynamic light scattering of KCPs and KSPs in ethanol with generated plots measuring Intensity %.

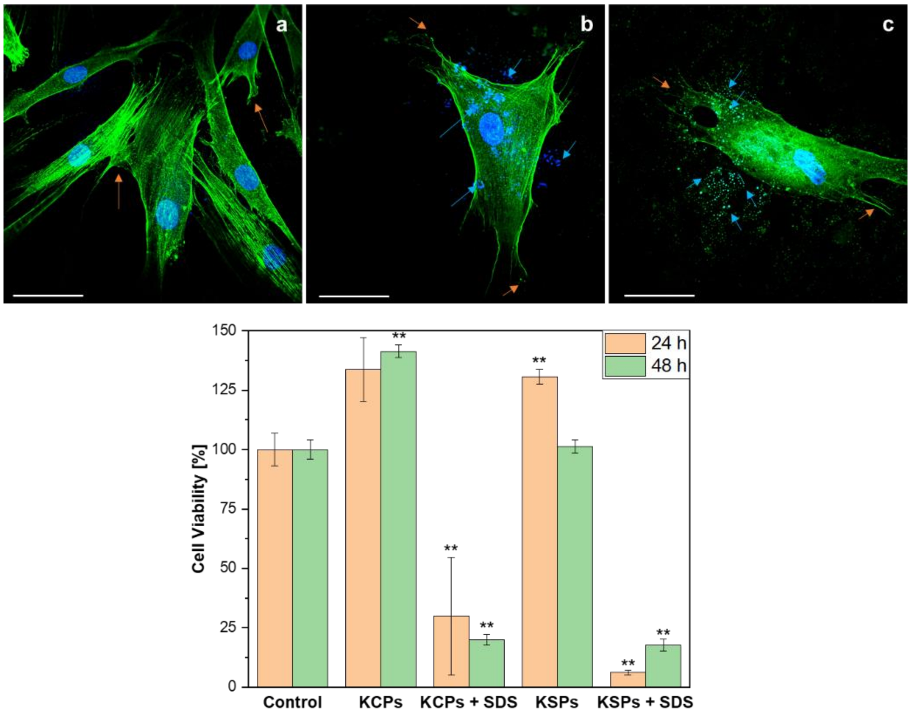

Figure 6S. HDFa cells cultured in the control experiments: (a) glass coverslips; (b) KCPs; (c) KSPs. Drops of KPs suspensions at a concentration of 1 mg/ml were deposited onto the glass coverslips to prepare control, un-patterned samples for cell growth. The particles are visible thanks to their autofluorescence signal in the blue channel (blue arrows). In all the samples under study, the cells presented a well spread-out morphology and no specific directionality of adhesion/growth was visible. Orange arrows indicate the presence of focal adhesion complexes as a result of the vinculin staining. The DAPI (blue) channel highlights the cells' nuclei, while the AF 488 (green) channel is used to image both the cytoskeleton (actin fibers) and the focal adhesion complexes of the cells. Scale bar 50 µm. (d) Cell viability results via MTS assay on HDFa cells, grown in the presence of the extraction media obtained after the incubation of 1 mg/ml of KPs for 24 and 48 h. For this experiment, all the KPs under study were considered, either fabricated by cysteine or sulfitolysis and in the presence or the absence of the surfactant SDS. A Student's *t*-test was conducted considering a $p < 0.01$ (**).

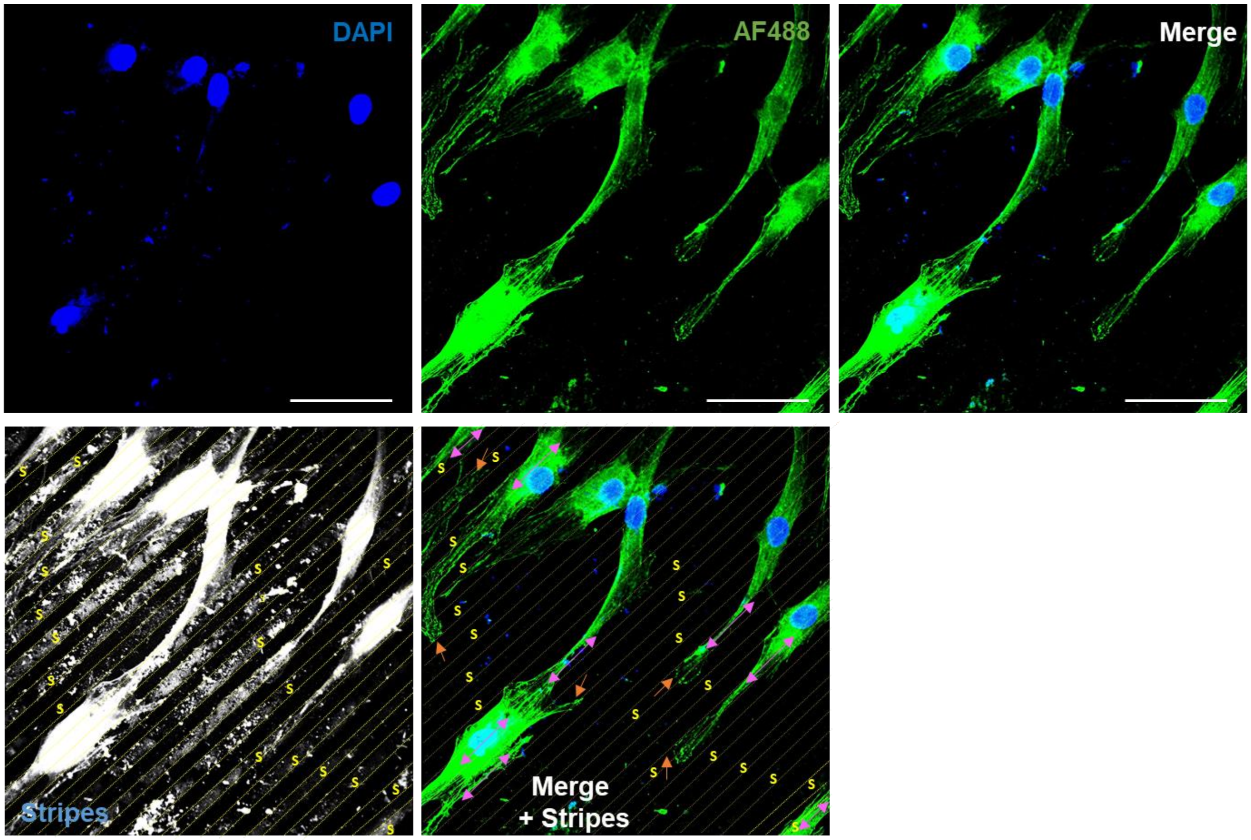

Figure 7S. HDFa cells onto 5-10 KCPs stripes. The stripes were visualized by leveraging on the KPs auto-fluorescence signal in the blue channel (white false coloring), and then their contour was overlaid onto the merged image. The locations of the stripes are indicated by the letter "S" (in correspondence of the 10 μm-wide area). Orange arrows indicate the presence of focal adhesion complexes as a result of the vinculin staining. Pink arrows indicate the preferential direction of growth for the stretched, adherent cells. The DAPI (blue) channel highlights the cells' nuclei, while the AF 488 (green) channel is used to image both the cytoskeleton (actin fibers) and the focal adhesion complexes of the cells. Scale bar 50 μm.

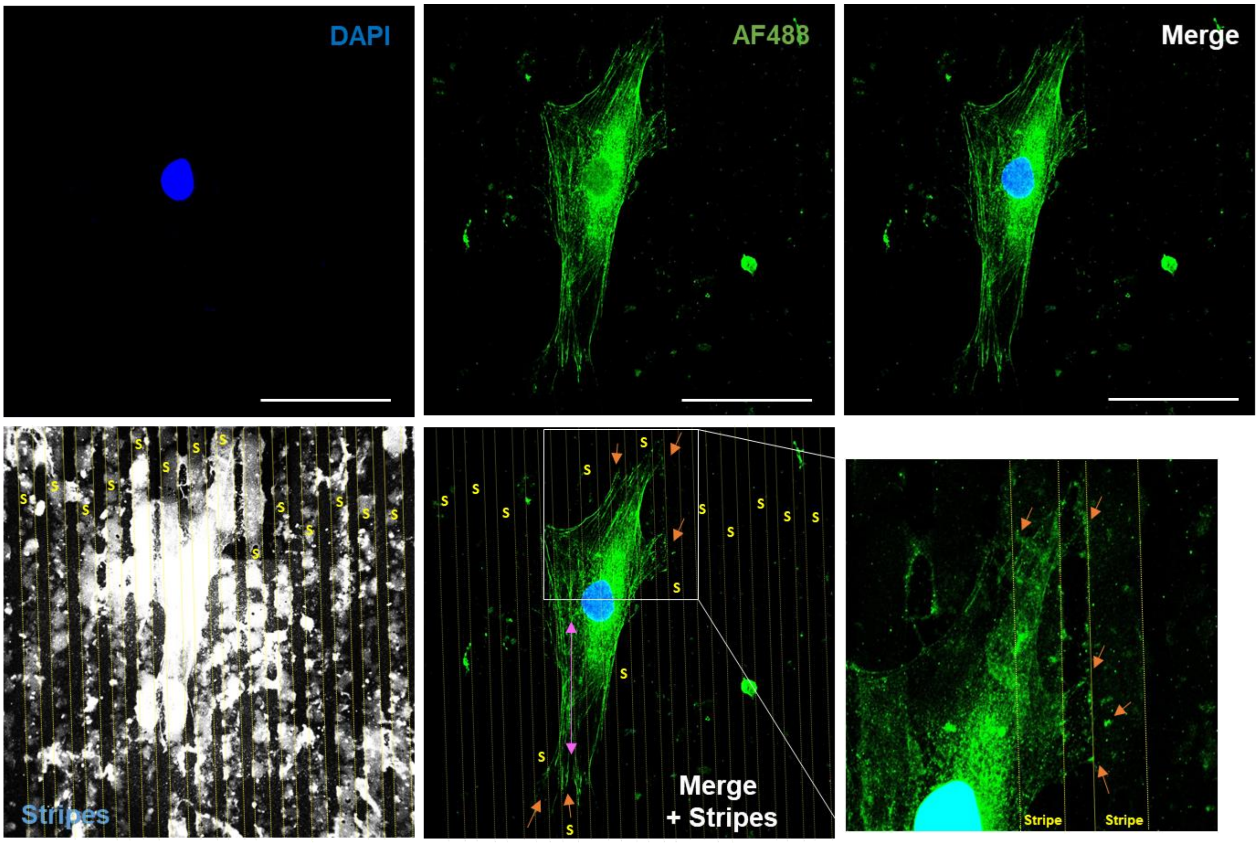

Figure 8S. HDFa cells onto 5-10 KCPs stripes. The stripes were visualized by leveraging on the KPs auto-fluorescence signal in the blue channel (white false coloring), and then their contour was overlaid onto the merged image. The locations of the stripes are indicated by the letter "S" (in correspondence of the 10 µm-wide area). Orange arrows indicate the presence of focal adhesion complexes as a result of the vinculin staining. Pink arrows indicate the preferential direction of growth for the stretched, adherent cell. The DAPI (blue) channel highlights the cell nucleus, while the AF 488 (green) channel is used to image both the cytoskeleton (actin fibers) and the focal adhesion complexes of the cell. Scale bar 50 µm.

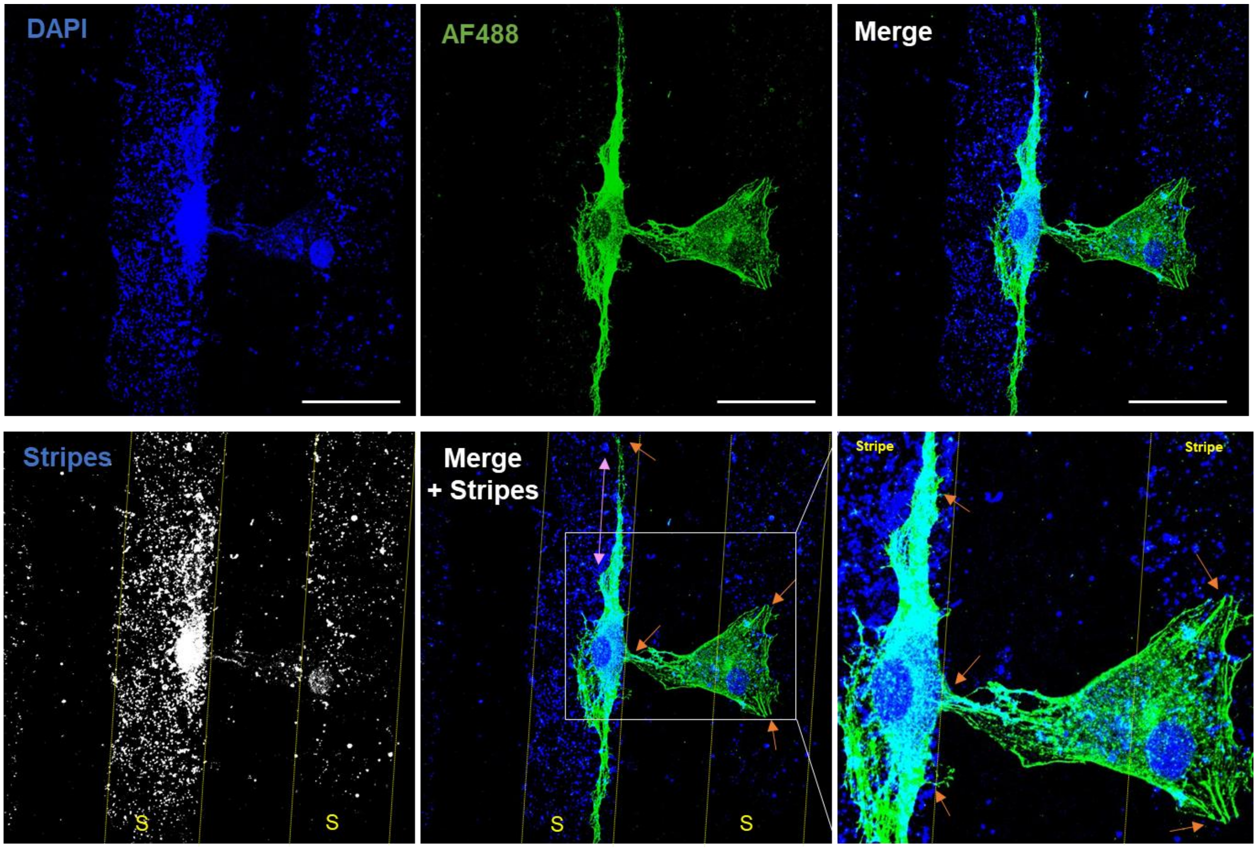

Figure 9S. HDFa cells onto 50-50 KCPs stripes. The stripes were visualized by leveraging on the KPs auto-fluorescence signal in the blue channel (white false coloring), and then their contour was overlaid onto the merged image. The locations of the stripes are indicated by the letter “S”. Orange arrows indicate the presence of focal adhesion complexes as a result of the vinculin staining. Pink arrows indicate the preferential direction of growth for the stretched, adherent cells. The DAPI (blue) channel highlights the cells’ nuclei, while the AF 488 (green) channel is used to image both the cytoskeleton (actin fibers) and the focal adhesion complexes of the cells. Scale bar 50 μm.

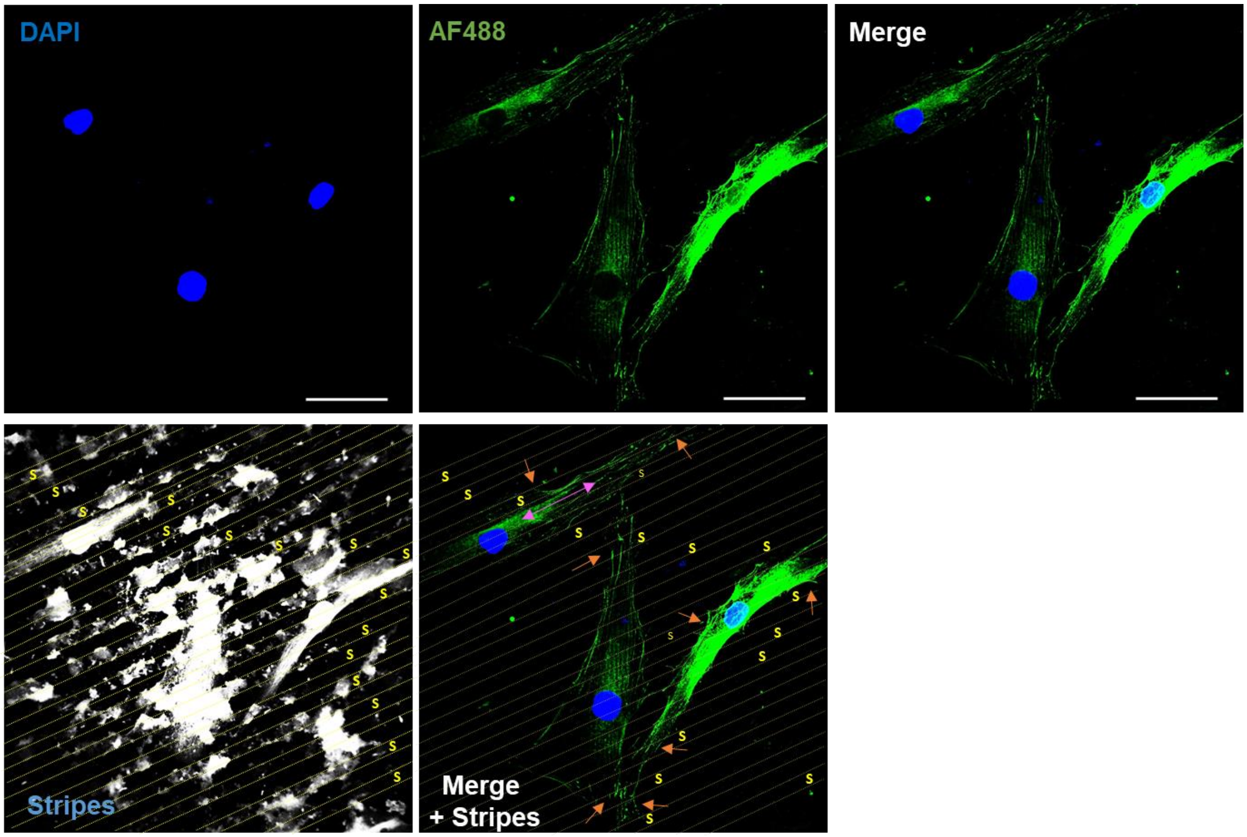

Figure 10S. HDFa cells onto 5-10 KSPs stripes. The stripes were visualized by leveraging on the KPs auto-fluorescence signal in the blue channel (white false coloring), and then their contour was overlaid onto the merged image. The locations of the stripes are indicated by the letter "S" (in correspondence of the 10 µm-wide area). Orange arrows indicate the presence of focal adhesion complexes as a result of the vinculin staining. Pink arrows indicate the preferential direction of growth for the stretched, adherent cells. The DAPI (blue) channel highlights the cells' nuclei, while the AF 488 (green) channel is used to image both the cytoskeleton (actin fibers) and the focal adhesion complexes of the cells. Scale bar 50 µm.

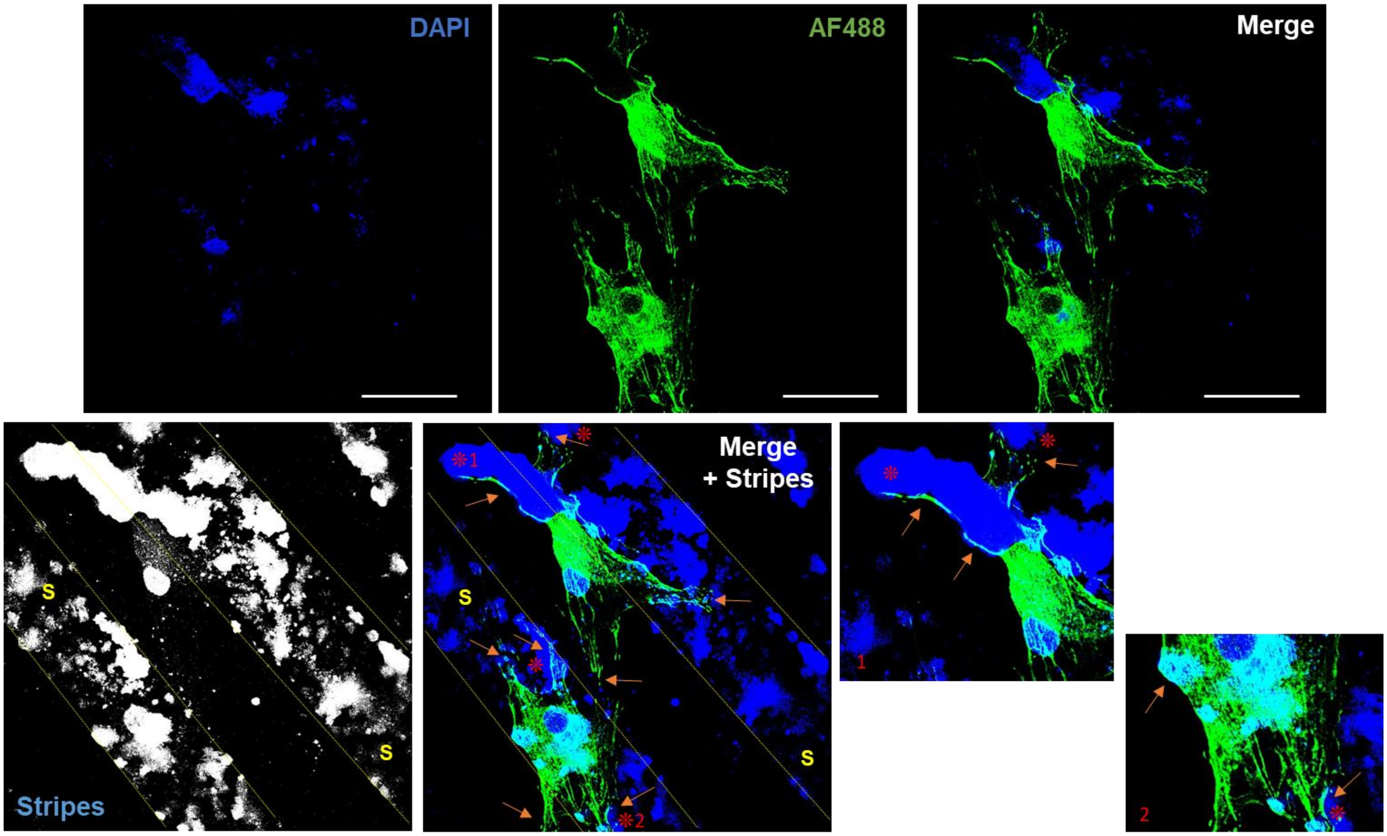

Figure 11S. HDFa cells onto 50-50 KSPs stripes. The stripes were visualized by leveraging on the KPs auto-fluorescence signal in the blue channel (white false coloring), and then their contour was overlaid onto the merged image. The locations of the stripes are indicated by the letter "S". Orange arrows indicate the presence of focal adhesion complexes as a result of the vinculin staining. Red asterisks underline the correspondence between the cell adhesion complex/cell membrane protrusion and the KSPs aggregates, as visible in insets (1) and (2). The DAPI (blue) channel highlights the cells' nuclei, while the AF 488 (green) channel is used to image both the cytoskeleton (actin fibers) and the focal adhesion complexes of the cells. Scale bar 50 µm.
